# Triglyceride-derived fatty acids reduce autophagy in a model of retinal angiomatous proliferation

**DOI:** 10.1172/jci.insight.154174

**Published:** 2022-03-22

**Authors:** Emilie Heckel, Gael Cagnone, Tapan Agnihotri, Bertan Cakir, Ashim Das, Jin Sung Kim, Nicholas Kim, Geneviève Lavoie, Anu Situ, Sheetal Pundir, Ye Sun, Florian Wünnemann, Kerry A. Pierce, Courtney Dennis, Grant A. Mitchell, Sylvain Chemtob, Flavio A. Rezende, Gregor Andelfinger, Clary B. Clish, Philippe P. Roux, Przemyslaw Sapieha, Lois E.H. Smith, Jean-Sébastien Joyal

**Affiliations:** 1 Department of Pharmacology, University of Montreal, Montreal, Quebec, Canada.; 2 Department of Pharmacology and Therapeutics, McGill University, Montreal, Quebec, Canada.; 3 Department of Ophthalmology, Boston Children’s Hospital, Harvard Medical School, Boston, Massachusetts, USA.; 4 Department of Pathology and Cell Biology, Institute for Research in Immunology and Cancer (IRIC), and; 5 Department of Pediatrics, University of Montreal, Montreal, Quebec, Canada.; 6 Metabolomics Platform, Broad Institute of MIT and Harvard University, Cambridge, Massachusetts, USA.; 7 Department of Ophthalmology, University of Montreal, Montreal, Quebec, Canada.

**Keywords:** Ophthalmology, Vascular Biology, Autophagy, Fatty acid oxidation, Retinopathy

## Abstract

Dyslipidemia and autophagy have been implicated in the pathogenesis of blinding neovascular age-related macular degeneration (NV-AMD). VLDL receptor (VLDLR), expressed in photoreceptors with a high metabolic rate, facilitates the uptake of triglyceride-derived fatty acids. Since fatty acid uptake is reduced in *Vldlr*^–/–^ tissues, more remain in circulation, and the retina is fuel deficient, driving the formation in mice of neovascular lesions reminiscent of retinal angiomatous proliferation (RAP), a subtype of NV-AMD. Nutrient scarcity and energy failure are classically mitigated by increasing autophagy. We found that excess circulating lipids restrained retinal autophagy, which contributed to pathological angiogenesis in the *Vldlr*^–/–^ RAP model. Triglyceride-derived fatty acid sensed by free fatty acid receptor 1 (FFAR1) restricted autophagy and oxidative metabolism in photoreceptors. FFAR1 suppressed transcription factor EB (TFEB), a master regulator of autophagy and lipid metabolism. Reduced TFEB, in turn, decreased sirtuin-3 expression and mitochondrial respiration. Metabolomic signatures of mouse RAP-like retinas were consistent with a role in promoting angiogenesis. This signature was also found in human NV-AMD vitreous. Restoring photoreceptor autophagy in *Vldlr*^–/–^ retinas, either pharmacologically or by deleting *Ffar1*, enhanced metabolic efficiency and suppressed pathological angiogenesis. Dysregulated autophagy by circulating lipids might therefore contribute to the energy failure of photoreceptors driving neovascular eye diseases, and FFAR1 may be a target for intervention.

## Graphical abstract

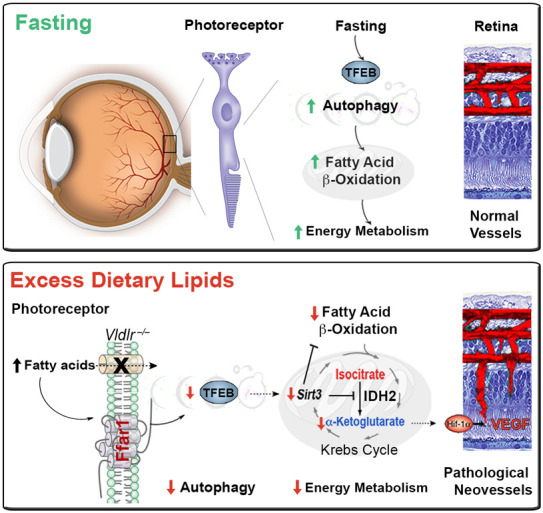

## Introduction

Neovascular age-related macular degeneration (NV-AMD) is the leading cause of vision loss in aging adults, and retinal angiomatous proliferation (RAP) occurs in 12%–15% of individuals with NV-AMD (1). Diseases of the macula, such as AMD, are likely to be more susceptible to dysregulations in energy metabolism. The macula contains the highest cone photoreceptor density with abundant mitochondria and high energy requirements (2, 3). Hence, a primary energy failure of photoreceptors was hypothesized to signal an increase in vascular supply to offset unmet neuronal energy demands in NV-AMD. Retinal neurons were classically believed to rely mainly on glucose to generate ATP (4, 5). We recently showed that fatty acids could also be oxidized by photoreceptors (6). The origin of these lipids is unknown, but a potential source is intracellular substrates controlled by autophagy. However, the adaptation of photoreceptors to nutrient scarcity or abundance regulated by autophagy has not been extensively studied (7). Although dyslipidemia (8) and disrupted autophagy (9, 10) have been previously associated with AMD, their role in the etiology of vascular disease has not been established.

Autophagy, or cellular cannibalism, is an evolutionary adaptation to starvation, improving metabolic efficiency by scavenging intracellular substrates used to fuel mitochondria or rebuild cell components (11). Recycling defective proteins and organelles is also a critical housekeeping role of autophagy, particularly in postmitotic neurons (12). Autophagy shapes the development of the visual system (13). Conditional deletion of autophagy-related genes (*Atg*) in photoreceptors disrupts mitochondrial volume and structure, leading to photoreceptor cell death and vision loss (14–16). In retinal pigment epithelial (RPE) cells, which line the posterior segment of the eye, autophagy recycles visual retinoids shed by photoreceptors to support vision (17). Finally, dysregulated RPE autophagy in patients with AMD results in metabolite changes (9, 10, 18). Hence, autophagy sustains vision and likely contributes to retinal energy metabolism, but the mechanisms disrupting autophagy in eye diseases, such as AMD, remain to be elucidated.

Autophagy of lipids, or macrolipophagy (19), is stimulated by starvation to provide energy by utilizing cytosolic lipid stores. Conversely, excess lipid impedes autophagy, giving rise to a phenotype reminiscent of metabolic syndrome (20, 21). Nutrient sensors that regulate autophagy have been identified for glucose and amino acids but not lipids (22, 23). Free fatty acid receptor 1 (FFAR1; GPR40) is expressed in pancreatic beta cells (24), the CNS, and the retina (25). Importantly, FFAR1 is a low-affinity receptor activated by micromolar concentrations of medium- and long-chain fatty acids (25), suggesting that it may be particularly active following loading, such as a lipid-rich meal. FFAR1 is, therefore, an ideal sensor of lipid surges, which may signal to curtail autophagy and oxidative metabolism when nutrient supply exceeds the metabolic needs of photoreceptors.

Lipid metabolism and autophagy are regulated by transcription factor EB (TFEB) (26). TFEB governs a transcriptional network (CLEAR network) that promotes both lysosomal biogenesis (27) and fatty acid β-oxidation (26, 28). TFEB is retained in the cytoplasm and inactivated when phosphorylated. In the presence of nutrients, mTORC1 forms a major kinase complex that phosphorylates and inactivates TFEB. Conversely, nutrient scarcity sensed by mTORC1 suppresses TFEB phosphorylation and allows calcineurin to dephosphorylate TFEB, which then translocates to the cell nucleus to initiate autophagy (27, 29). Calcineurin is a calcium-dependent phosphatase highly expressed in the retina (30), working downstream and independently of mTORC1. Even during starvation, depletion of calcineurin restrains TFEB activity and autophagy (31). Although the mechanism regulating calcineurin activity is only partially understood, phosphorylation of serine 197 was shown to decrease its phosphatase activity (32–34). Hence, nutrient scarcity triggers a transcriptional program governed by TFEB to produce energy from lipids and autophagy, which may be important in the retina.

Here, we examined the hypothesis that autophagy provides an alternative fuel source for photoreceptors during nutrient scarcity and is abrogated by high circulating lipid (lipid loading). The VLDL receptor (VLDLR) facilitates the uptake of fatty acids in tissues capable of lipid β-oxidation, such as the heart and retina (35). To explore the role of elevated levels of circulating lipids on retinal autophagy and energy metabolism, we used *Vldlr-*deficient *(Vldlr*^–/–^) mice that had reduced fatty acid uptake into cells and high circulating triglyceride-derived lipid levels. Murine retinas do not form a macula, a significant limitation of all NV-AMD murine models. Still, *Vldlr*^–/–^ photoreceptors are energy deficient and drive the formation of retinal vascular lesions reminiscent of RAP, a subtype of neovascular AMD (6, 36) and later choroidal neovascularization (36). We showed that high levels of extracellular lipids detected by FFAR1 in photoreceptors could suppress TFEB activity and autophagy, sirtuin-3 levels, and mitochondrial respiration. The abundance of circulating lipids from decreased uptake in *Vldlr*^–/–^ mice triggered a decline in autophagy that exacerbated the energy failure of photoreceptors, stimulating VEGFA production to drive compensatory albeit pathological angiogenesis. Autophagy, therefore, provides a source of fuel for mitochondrial respiration in photoreceptors and may be suppressed by high circulating lipids contributing to neovascular eye diseases.

## Results

### Autophagy is suppressed in the Vldlr^–/–^ mouse model of RAP-like neovascularization.

**Figure 1:**
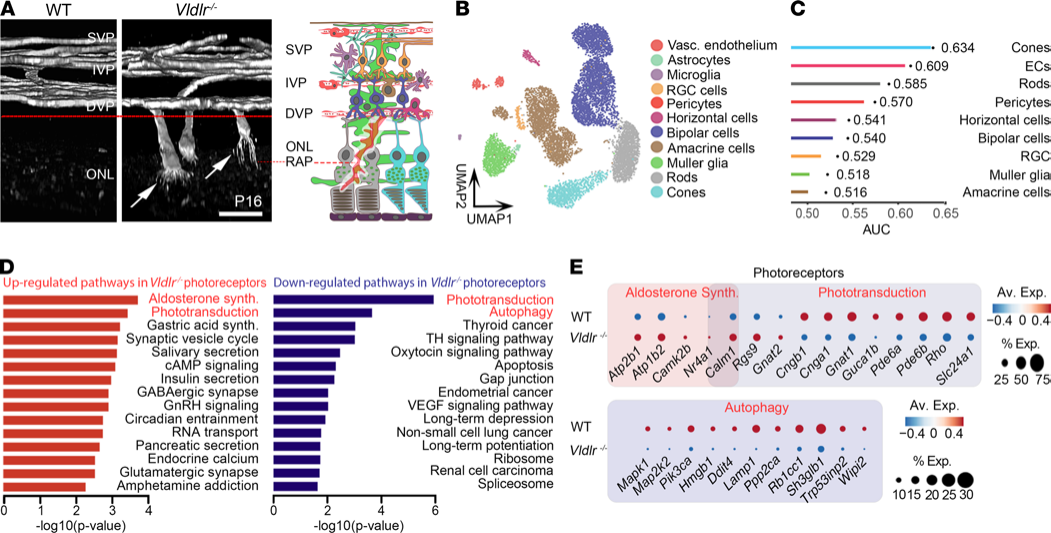
Phototransduction and autophagy are dysregulated in the murine *Vldlr^–/–^* RAP model. (**A**) 3D confocal reconstruction of lectin-stained WT and *Vldlr^–/–^* retina with retinal angiomatous proliferation (RAP, white arrows). The red line delineates the inner (above) and outer retina (below, photoreceptors and RAP-like lesions). Vascular plexus (VP); SVP, superficial VP; IVP, intermediate VP; DVP, deep VP; ONL, outer nuclear layer. Scale bars: 20 μm. (**B**) UMAP representation of retinal cell types identified by single-cell RNA-Seq from P14 WT and *Vldlr^–/–^* retinas, preceding the peak of RAP-like neovascular lesion formation at P16. *n* = 3801 WT and 5642 *Vldlr^–/–^* cells pooled from 3 retinas per group. (**C**) Cell-type prioritization using Augur to represent the extent of transcriptomic perturbations associated with the RAP-like *Vldlr*^–/–^ phenotype compared with WT (AUC for the random forest classifier performance on each cell type). (**D**) Enriched biological pathways from either upregulated or downregulated genes (adjusted *P* < 0.05) in *Vldlr^–/–^* photoreceptors. (**E**) Dot plot of the gene transcripts driving the top 2 most dysregulated pathways (**D**) in *Vldlr^–/–^* photoreceptors compared with WT. See also Supplemental Figures 1 and 2.

**Figure 2:**
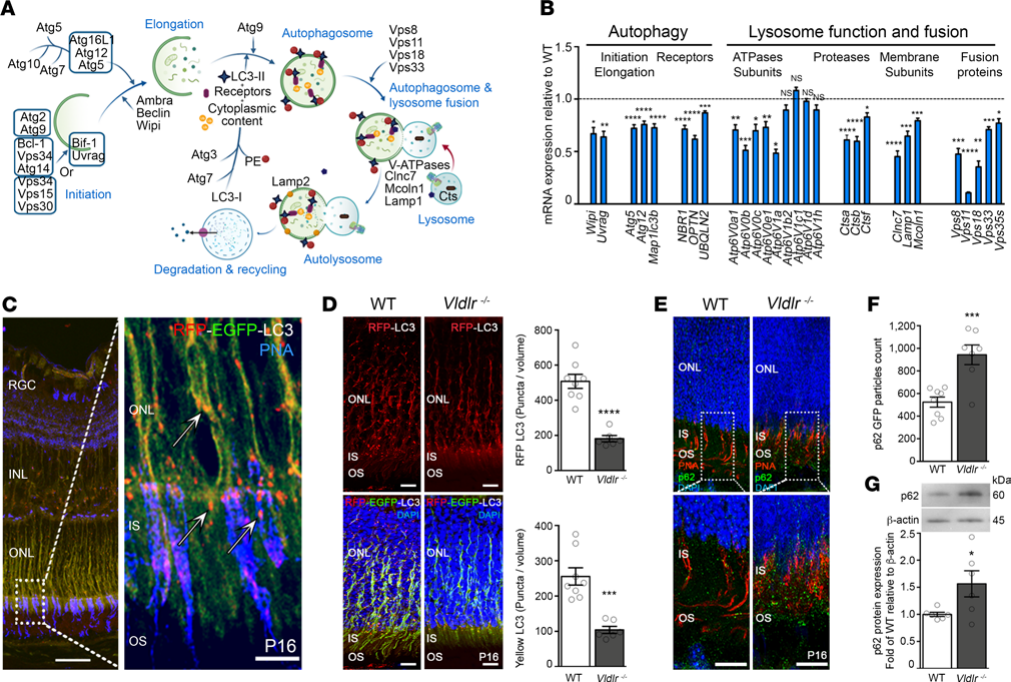
Autophagy is suppressed in *Vldlr^–/–^* photoreceptors. (**A**) Schematic representation of autophagy and (**B**) related mRNA gene expression in *Vldlr^–/–^* retinas compared with WT. *n* = 12–30 retinas, P14. (**C**) CAG-RFP-EGFP-LC3 expression reports autophagy flux (white arrow). Peanut agglutinin (PNA) labels cone photoreceptors. Scale bars: 5 μm (left) and 10 μm (right). (**D**) Autophagy flux was curtailed in CAG-RFP-EGFP-LC3/*Vldlr^–/–^* mouse. *n* = 6–8 retinas. Scale bars: 10 μm. (**E** and **F**) Representative immunofluorescence and (**G**) immunoblot showing p62 accumulation in *Vldlr^–/–^* (*n* = 10–14) retinas. Scale bars: 10 μm. RGC, retinal ganglion cell; INL and ONL, inner and outer nuclear layer; IS and OS, photoreceptor inner and outer segment. Data are represented as mean ± SEM. *P < 0.05, **P < 0.01, ***P < 0.001, ****P < 0.0001. One-way ANOVA with Tukey’s multiple-comparison test and 2-tailed Student’s *t* test.

We previously showed that *Vldlr*^–/–^ mice retinas develop RAP-like neovascular lesions that invade the energy-deficient photoreceptor outer nuclear layer (Figure 1A) (6). To unbiasedly explore the cause of this energy deficit in *Vldlr*^–/–^ retinas, we characterized the heterogeneity of individual retinal cells using single-cell transcriptomics (37). We clustered and annotated individual retinal cell types using known gene markers (Figure 1B and Supplemental Figure 1; supplemental material available online with this article; https://doi.org/10.1172/jci.insight.154174DS1). Next, we prioritized the cell types most responsive to biological perturbations in our single-cell data, using a recently published machine-learning framework called Augur (38). Photoreceptors, both cones and rods, as well as endothelial cells, were identified by machine learning as the top 3 most perturbed cell types in the *Vldlr*^–/–^ neovascular RAP model (Figure 1C). Interestingly, *VLDLR* expression was predominantly detected in photoreceptors (cones more than rods), both in mice and human retinas (39) by single-cell transcriptomics (Supplemental Figure 2, A and B). We therefore focused our analysis on the *Vldlr^–/–^*-induced transcriptomic perturbations in photoreceptors. Among the differentially expressed genes (DEGs) in *Vldlr^–/–^* photoreceptors (103 upregulated genes, 626 downregulated genes, adjusted *P* < 0.05, Supplemental Table 1), aldosterone synthesis, phototransduction, and autophagy were the most enriched pathways in this RAP model (Figure 1D). Dot plot representation of the DEGs underlying these pathways highlighted the important downregulation of autophagy-related genes in *Vldlr*^–/–^relative to WT photoreceptors (Figure 1E). Moreover, gene set enrichment analysis (GSEA) for autophagosome formation was significantly suppressed in *Vldlr*^–/–^compared with WT photoreceptors (Supplemental Figure 2C). We next dissected the transcriptional signature of key autophagy genes in *Vldlr*^–/–^ retinas compared with the control (Figure 2A). Interestingly, we observed a global reduction in genes critical to autophagy initiation and elongation; in autophagy-linked receptors; and in genes implicated in lysosomal function and fusion, such as v-ATPases subunits, lysosomal proteases, membrane subunits, and vacuolar fusion proteins (Figure 2B). Hence, we unbiasedly identified autophagy as one of the most transcriptionally downregulated pathways in *Vldlr*^–/–^ photoreceptors.

We then used the autophagy reporter mouse CAG-RFP-EGFP-LC3 to characterize autophagy flux in the mouse retina. *Map1lc3b* (or LC3B) is a marker of autophagosome membrane expansion and fusion events, and dual fluorescent labeling tracks phagocytic compartments based on their acidity. Combined EGFP and red fluorescent protein (RFP) fluorescence yields a yellow signal within autophagosomes. After the fusion of autophagosomes with acidic lysosomes, the EGFP signal is quenched while the more resistant RFP signal persists. Autophagy flux can be measured by comparing the relative expression of yellow (autophagosomes) and red fluorescence (autolysosomes) in autophagy reporter mice (Supplemental Video 1). In line with our single-cell transcriptomics results, higher autophagy flux colocalized with photoreceptors in reporter mice (Figure 2C). We crossed the *Vldlr*^–/–^ mouse with the CAG-RFP-EGFP-LC3 reporter mouse to measure autophagy flux in our murine RAP model. Compared with control retinas, autophagy flux was reduced by more than 60% in *Vldlr*^–/–^ retinas (Figure 2D). Moreover, p62/SQSTM1 protein that is generally degraded by autophagy was found to accumulate in *Vldlr*^–/–^ retinas, mostly in photoreceptor inner and outer segments by immunofluorescence (Figure 2, E and F) and by Western blot (Figure 2G). These results confirmed that autophagy flux was reduced in photoreceptors in RAP-like *Vldlr*^–/–^retinas.

### Triglyceride-derived lipids curb autophagy in photoreceptors.

**Figure 3:**
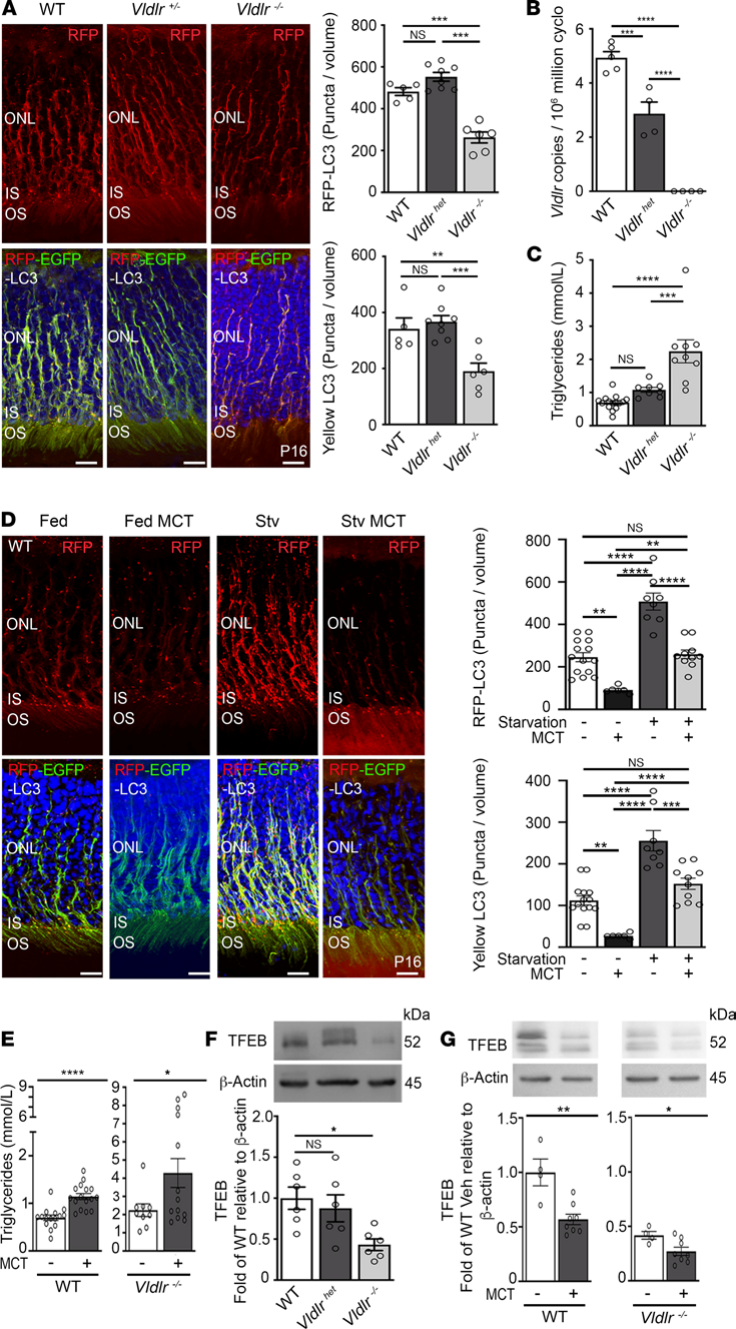
Triglyceride-derived lipids curb autophagy in photoreceptors. (**A**) CAG-RFP-EGFP-LC3 autophagy flux colocalization and quantification of starved P16 WT (*n* = 5), *Vldlr* heterozygotes (*n* = 8), and mutant mice (*n* = 6). Scale bars: 10 μm. (**B** and **C**) *Vldlr* mRNA retinal expression, serum triglyceride concentration in WT (*n* = 5–7), *Vldlr^+/–^* heterozygotes (het, *n* = 4–8), and *Vldlr^–/–^* mice (*n* = 4–13). (**D**) Starvation (8 h) increased autophagy flux in CAG-RFP-EGFP-LC3 (WT) mice, which was curbed by a lipid load with MCT (*n* = 6–14). Scale bars: 10 μm. (**E**) Serum triglyceride concentrations of starved P16 WT and *Vldlr^–/–^* mice gavaged with MCT or vehicle (*n* = 9–16). (**F**) TFEB expression in WT, *Vldlr*^+/–^ (het), and *Vldlr^–/–^* retinas (*n* = 6 per condition). (**G**) Retinal TFEB expression was suppressed by MCT in WT mice and more so in *Vldlr^–/–^* mice (Veh; *n* = 4 experiments, MCT; *n* = 8 experiments). Stv, starved mice; INL and ONL, inner and outer nuclear layer; IS and OS, photoreceptor inner and outer segment. Data are represented as mean ± SEM. *P < 0.05, **P < 0.01, ***P < 0.001, ****P < 0.0001. Two-tailed Student’s *t* test and 1-way ANOVA with Tukey’s or Dunnett’s multiple-comparison test.

We proceeded to examine possible causes of decreased autophagy in *Vldlr*^–/–^ photoreceptors. VLDLR facilitates the uptake of fatty acid in tissues capable of lipid β-oxidation, such as the retina, and we previously showed that long-chain fatty acids are fuel substrates of photoreceptors (6). We first assessed whether reduced VLDLR expression could impede fatty acid uptake by photoreceptors and directly affect autophagy. Unlike *Vldl*r^–/–^ mice, the heterozygous *Vldlr*^+/–^ mouse crossed with the CAG-RFP-EGFP-LC3 reporter mouse had preserved retinal autophagy flux similar to WT controls (Figure 3A), despite having reduced *Vldlr* expression levels (Figure 3B). Hence, the level of *Vldlr* expression did not directly affect retinal autophagy. We therefore hypothesized that excess circulating lipid nutrients in *Vldlr*^–/–^ mice could regulate autophagy in photoreceptors and influence the development of neovascular AMD. Indeed, *Vldlr*-deficient mice (Figure 3B) with elevated triglyceride levels (Figure 3C) also had decreased photoreceptor autophagy flux (Figure 3A) and developed pathological RAP-like neovessels (Figure 1A). Heterozygous *Vldlr*^+/–^ mice, with sufficient VLDLR receptors to maintain normal circulating triglyceride levels (Figure 3C), formed normal retinas devoid of pathological neovessels (6). These results are consistent with circulating triglyceride regulating retinal autophagy in RAP-like *Vldlr*^–/–^ retinas.

Lipids stimulate or curtail autophagy in the liver, depending on dose and duration of exposure (40), but how lipids regulate autophagy in the neural retina is undefined. We thus examined the effects of circulating lipid levels on retinal autophagy. Retinal autophagy flux was increased by starvation in WT mice (Figure 3D), suggesting that autophagy may participate in the metabolic adaptation of the retina to reduced nutrient availability. Contrarily, WT pups fed with medium-chain triglyceride oil (MCT, gavaged), whether starved or not prior to treatment, showed a decreased number of autophagosomes (yellow puncta; 40%, *P* <0.001) and autolysosomes (red puncta; 49%, *P* < 0.0001) in photoreceptors (Figure 3D). MCT oil is composed of mid- to long-chain fatty acids (C8-C16), comparable to the circulating lipid profile of *Vldlr*^–/–^ mice (6). MCT is readily absorbed and yields predictable and timely increases in circulating triglyceride levels in WT and *Vldlr*^–/–^ mouse pups (Figure 3E), analogous to those reported in humans (41). These results were consistent with circulating triglyceride regulating autophagy in RAP-like *Vldlr*^–/–^ retinas.

We next investigated how circulating lipids regulate autophagy. Lipid metabolism and autophagy are coregulated by TFEB (26), which governs a transcriptional network (CLEAR network) promoting lysosomal biogenesis (27) and fatty acid β-oxidation (26, 28). TFEB expression was suppressed in *Vldlr*^–/–^ mice (Figure 3F) with elevated triglyceride levels (Figure 3C) but not in heterozygous *Vldlr*^+/–^ mice with triglyceride levels comparable to WT controls. Moreover, treatment with MCT increased triglyceride levels in WT and *Vldlr*^–/–^ mice and suppressed retinal TFEB expression (Figure 3G). Hence, in contrast to starvation that increased autophagy, lipid loading suppressed TFEB expression and retinal autophagy.

### FFAR1 suppresses TFEB.

**Figure 4:**
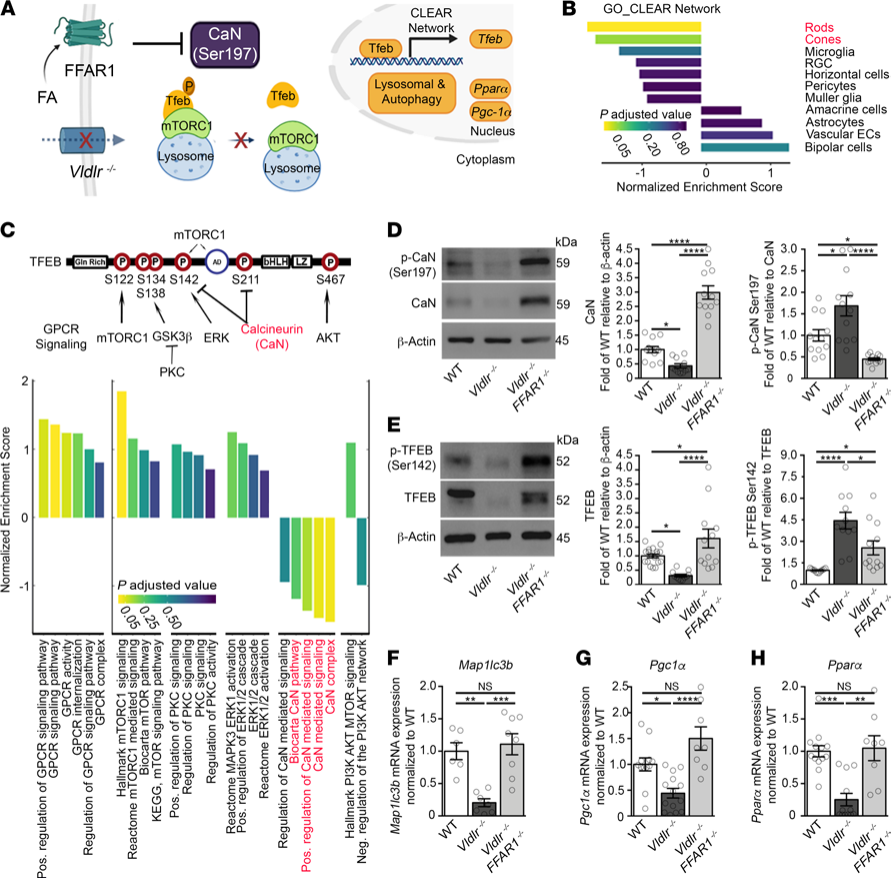
Free fatty acid receptor 1 suppresses TFEB. (**A**) Schematic representation of fatty acid signaling through FFAR1, modulating phosphatase activity of calcineurin (CaN) via serine 197 phosphorylation, which prevents the nuclear translocation of TFEB. (**B**) GSEA averages of normalized enrichment scores (NESs) for the GO_CLEAR Network gene set across retinal cell types in *Vldlr^–/–^* retinas relative to WT. (**C**) Schematic representation of known TFEB regulating pathways (top) and their corresponding NES (bottom) in *Vldlr^–/–^* photoreceptors relative to WT at P14 (**B** and **C**, *n* = 9000 cells pooled from 3 retinas per group). (**D**) Immunoblot quantifications of phosphorylated (serine 197), total CaN, and (**E**) phosphorylated (serine 142) and total TFEB in *Vldlr^–/–^* retinas (*n* = 22–24) compared with WT (*n* = 22–36) and *Vldlr^–/–^*/*Ffar1^–/–^* retinas (*n* = 20–24) at P16. (**F–H**) mRNA expression of *Map1lc3b,* *Pgc1a,* and *Ppara* in WT (*n* = 12–18), *Vldlr^–/–^* (*n* = 14–20), and *Vldlr^–/–^*/*Ffar1^–/–^* retinas (*n* = 8–16) at P16. Data are represented as mean ± SEM. *P < 0.05, **P < 0.01, ***P < 0.001, ****P < 0.0001. One-way ANOVA or Kruskal-Wallis test with Tukey’s or Dunn’s multiple-comparison test. See also Supplemental Figures 3 and 4.

We reasoned that a lipid cell membrane receptor might regulate TFEB activity and autophagy. We previously identified the presence of known lipid receptors in the retina (6), and among them, FFAR1 was abundant in photoreceptors by RNAscope in situ hybridization (Supplemental Figure 3A). Unbound free fatty acids detected by FFAR1 are liberated from circulating triglycerides by lipoprotein lipases at the cell surface (42). We therefore examined whether FFAR1 is a lipid sensor able to regulate autophagy and lipid metabolism through TFEB signaling in photoreceptors (Figure 4A). The downstream transcriptional network of TFEB (CLEAR network) was specifically repressed in *Vldlr*^–/–^ photoreceptors (Figure 4B). TFEB activity is determined by its phosphorylation; it is retained in the cytoplasm and inactive when phosphorylated. We then screened for upstream pathways known to phosphorylate TFEB and that were differentially expressed in *Vldlr*^–/–^ photoreceptors. Calcineurin signaling was strongly suppressed in *Vldlr^–/–^* photoreceptors by single-cell analysis of curated gene enrichment sets (Figure 4C). Calcineurin is a serine/threonine protein phosphatase that enables TFEB activity (31). Hence, we hypothesized that triglyceride-derived free fatty acids sensed by FFAR1 in photoreceptors signaled through calcineurin to suppress TFEB (Figure 4A).

To establish the causal role of FFAR1 in calcineurin and TFEB signaling, we measured calcineurin expression in *Vldlr*^–/–^ retinas compared with WT and *Ffar1*-deficient *Vldlr*^–/–^ mice. We observed a significant reduction in total calcineurin expression (57%, *P* < 0.05) and a relative increase in calcineurin phosphorylation at serine 197 (69%, *P* < 0.05) (Figure 4D). Calcineurin phosphatase activity was previously shown to be attenuated by phosphorylation of serine 197 (32). Moreover, changes in calcineurin expression mirrored a corresponding reduction in TFEB protein expression (66.4%, *P* < 0.05) and a 5-fold increase in relative TFEB S142 phosphorylation in *Vldlr*^–/–^ retinas (Figure 4E). Both calcineurin and TFEB levels were rescued by deleting *Ffar1* in *Vldlr*^–/–^ mice (Figure 4, D and E), in contrast to other upstream TFEB signaling pathways we examined (Supplemental Figure 3, B–F). Finally, key TFEB transcriptional targets were also rescued in double knockout *Vldlr*^–/–^/*Ffar1^–/–^* retinas, such as autophagy markers ATG5 and *Map1lc3b* (Figure 4F and Supplemental Figure 3, G and H), and the expression of *Pgc1a* and *Ppara* (Figure 4, G and H), essential for mitochondrial biogenesis and lipid metabolism. These results suggest a causal relationship between FFAR1 and TFEB activity.

In vivo observations were then confirmed in vitro using 661W cells derived from cone photoreceptors with some retinal ganglion cell properties (43). FFAR1 lipid agonist palmitate decreased total calcineurin expression and increased its phosphorylation (serine 197) in 661W cells, which were rescued by silencing *Ffar1* (Supplemental Figure 4, A and B). Since TFEB modulates its own expression, we generated a stable 661W cell line containing a *Tfeb* promoter driving a luciferase reporter gene. MCT treatment reduced TFEB transcriptional activity in reporter cells, which was restored by silencing *Ffar1* (Supplemental Figure 4C). When starved, TFEB translocated to the cell nucleus to increase autophagy (Supplemental Figure 4, D and E), which was prevented by treating 661W cells with FFAR1 agonists (GW9508 and MCT; Supplemental Figure 4, D and E). Indeed, more TFEB was retained in the cytoplasm of GW9508-treated cells than in the corresponding nuclear fractions (Supplemental Figure 4F). Hence, a lipid load sensed by FFAR1 also prevented the nuclear translocation of TFEB and curbed autophagy in 661W cells.

### Human vitreous and mouse retina with RAP phenotypes share a common metabolite signature.

**Figure 5:**
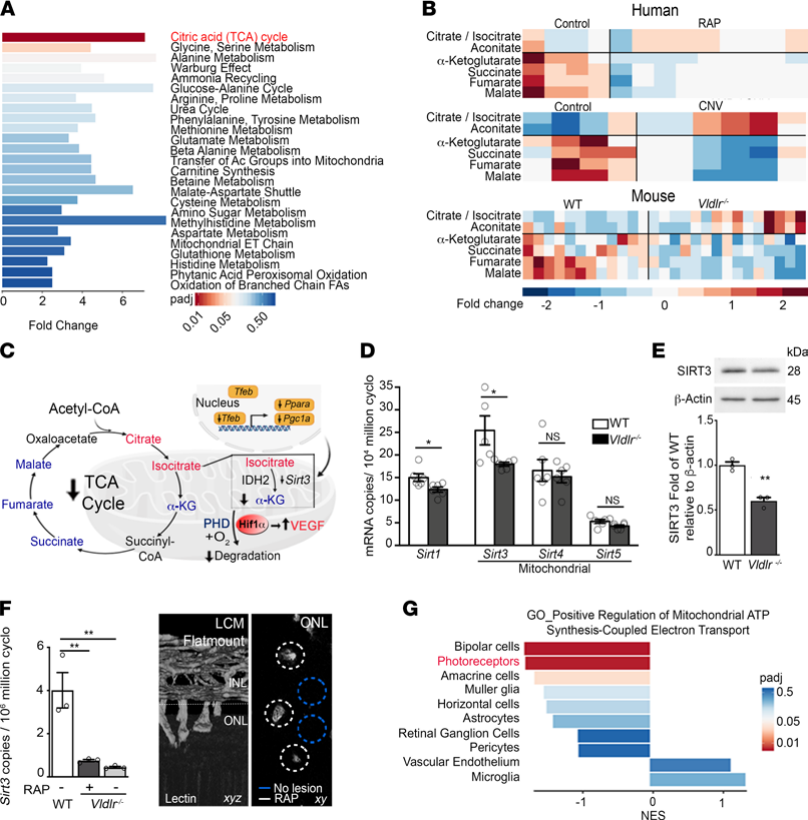
Human and mouse with RAP phenotypes share a common metabolite signature. (**A**) Metabolomics pathway enrichment analysis of differentially regulated metabolites from mouse *Vldlr^–/–^* retinas (*n* = 15) and human RAP vitreous (*n* = 9) compared with their respective control (WT, *n* = 12; and macular hole, *n* = 4). Ac, acetyl; ET, electron transport. (**B**) Heatmap of TCA cycle metabolites (**A**) of *Vldlr^–/–^* retinas and human RAP and CNV vitreous relative to their respective controls. *n* = 4 (control), 6 (CNV), and 9 (RAP) human vitreous; 12 (WT) and 15 (*Vldlr*^–/–^) retinas. (**C**) Schematic representation of TCA cycle perturbation hypothesized to contribute to pathological angiogenesis in RAP. IDH2 enzymatic activity is governed by SIRT3. Lower α-KG levels stabilize HIF1α and stimulate VEGFA secretion, increasing vascular supply. (**D**) *Sirt1* and mitochondrial sirtuin (*Sirt3*, *Sirt4,* and *Sirt5*) mRNA retinal expression at P16. *n* = 12 retinas per group. (**E** and **F**) SIRT3 protein expression (**E**, *n* = 6 retinas) measured in whole retinas, and (**F**) mRNA expression in photoreceptors obtained by laser capture microdissection (LCM) of RAP-like lesions (white circles) and adjacent regions (blue circles) of WT and *Vldlr*^–/–^ retinas by qRT-PCR (*n* = 6 retinas per group) at P16. Retinal flat mounts were sectioned across the photoreceptor outer nuclear layer (ONL; below the dotted line, left). INL, inner nuclear layer. (**G**) Normalized enrichment scores (NESs) for the GO_Positive Regulation of Mitochondrial ATP Synthesis Coupled Electron Transport gene set from GSEA of differentially expressed genes between *Vldlr^–/–^* and WT across retinal cell types in *Vldlr^–/–^* retinas relative to WT. RGCs, retinal ganglion cells; ECs, endothelial cells. *n* = 3801 WT and 5642 *Vldlr^–/–^* cells pooled from 3 retinas per group. Data are represented as mean ± SEM. *P < 0.05, **P < 0.01. One-way ANOVA with Tukey’s multiple-comparison test and 2-tailed Student’s *t* test. See also Supplemental Figure 5.

Since autophagy helps sustain energy homeostasis in many starved tissues (22), reduced autophagy in the *Vldlr*^–/–^ retina may contribute to the metabolic deficiency associated with the RAP phenotype. Previous work showed that changes in photoreceptors’ activity could be reflected in the vitreous proteome (44). We therefore analyzed the metabolomics profile of vitreous from study participants with NV-AMD, both RAP and choroidal neovascular disease (CNV), and control participants with macular hole (Supplemental Table 2). We then compared human metabolite profiles to mouse *Vldlr*^–/–^ retinas. Human and mouse metabolite profiles were closely correlated by unsupervised principal component (PC) analysis (PC2 and PC3; Supplemental Figure 5A) once we excluded the differences accounted by species (PC1; Supplemental Figure 5B). We detected 223 annotated metabolites in human RAP/CNV vitreous and 128 in *Vldlr^–/–^* retinas; 82 (30.4%) metabolites were detected in both human and mouse samples (Supplemental Figure 5C), with 37 sharing a distinctive signature (Supplemental Figure 5D). The citric acid cycle (TCA) was the most significantly dysregulated pathway of the shared metabolites from human RAP vitreous and mice *Vldlr*^–/–^ retinal samples (Figure 5A). We then focused our analysis on the TCA cycle; metabolites upstream of isocitrate dehydrogenase 2 (IDH2) accrued, while downstream metabolites such as α-ketoglutarate (α-KG) were depleted compared with controls (Figure 5B). Hence, the metabolite profile of human vitreous with neovascular AMD and *Vldlr*^–/–^ retinas with AMD-like RAP lesions were both suggestive of restricted TCA cycle flux at the level of IDH2 (Figure 5C), possibly contributing to decreased metabolic efficiency.

We then investigated how FFAR1 and TFEB could modulate the enzymatic activity of IDH2. TFEB drives the expression of PGC1α (Figure 4G), which in turn binds to the promoter region of sirtuin-3 *(Sirt3)* (45), regulating its expression (Figure 5C). Accordingly, *Tfeb* depletion in 661W cells suppressed TFEB, ATG5, LC3B-II, and SIRT3 protein expression and *Ppara* and *Pgc1a* mRNA expression (Supplemental Figure 6, A–C). SIRT3 is a mitochondrial NAD+ deacetylase and master regulator of mitochondrial energy metabolism (46, 47) targeting IDH2 (48) (Figure 5C). SIRT3 was abundant in the WT retina compared with other sirtuins, yet both its mRNA and protein expression were diminished in *Vldlr*^–/–^ retinas (Figure 5, D and E), specifically in laser microdissected photoreceptors (Figure 5F). We also found a significant reduction in a gene set expression regulating mitochondrial ATP synthesis–coupled electron transport in *Vldlr*^–/–^ photoreceptors (Figure 5G). Moreover, FFAR1 agonists prevented *Sirt3* expression in starved 661W cells without significant compensation from *Sirt1* or other mitochondrial sirtuins (*Sirt4* and *Sirt5*) (Supplemental Figure 6D). In line with this hypothesis, MCT treatment increased IDH2 acetylation (Supplemental Figure 6E), which is reported to hinder its enzymatic activity (48). A reduction in α-KG was previously shown to stabilize HIF1α and increase VEGFA secretion (6), which drove pathological angiogenesis (Figure 1A). *Ffar1* depletion in *Vldlr*^–/–^ retinas rescued HIF1α stabilization and VEGFA secretion (6). Together, these data suggested a rationale for the TCA cycle metabolite signature contributing to neovascularization in RAP.

### Ffar1 regulates oxidative metabolism and pathological angiogenesis.

**Figure 6:**
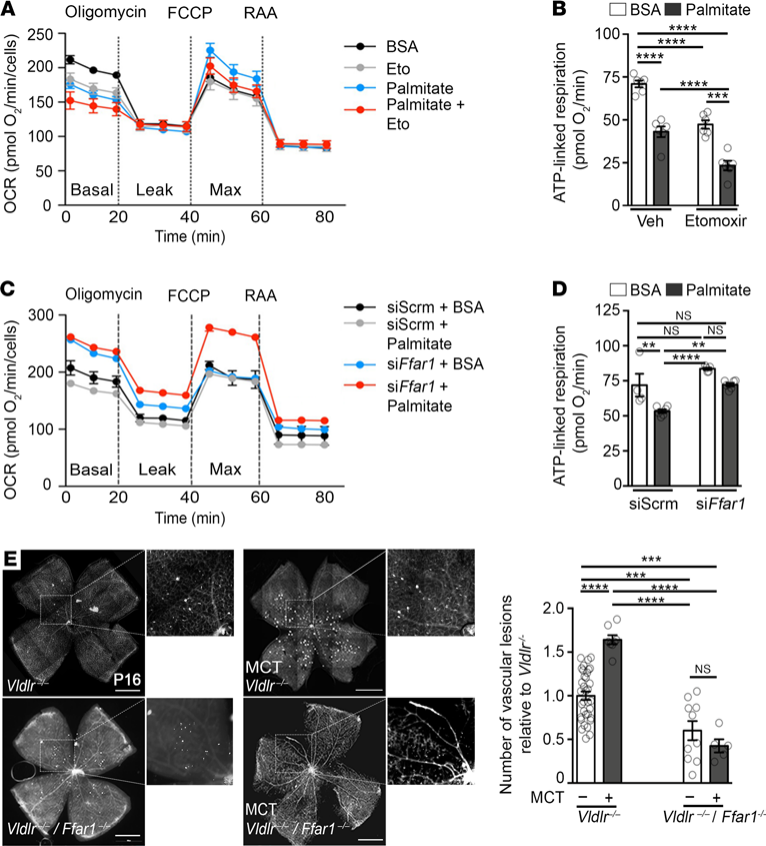
Ffar1 regulates oxidative metabolism and pathological angiogenesis. (**A–D**) Oxygen consumption rate (OCR) and ATP-linked respiration of 661W cells exposed to palmitate conjugated to BSA (8 hours, 0.2 mM) or control (BSA alone), measured by Seahorse analyzer, in the presence or absence of fatty acid β-oxidation inhibitor (**A** and **B**), etomoxir (40 μM), and in cells transfected with scrambled or *Ffar1* siRNA (**C** and **D**). *n* = 5–6 experiments per group. (**E**) Quantification of RAP-like vascular lesions (white spots) in *Vldlr^–/–^* and *Vldlr^–/–^*/*Ffar1^–/–^* retinas of mice fed with MCT (*n* = 5–8 retinas) or not (*n* = 10–34 retinas) at P16. Scale bars: 1 mm. Data are represented as mean ± SEM. *P < 0.05, **P < 0.01, ***P < 0.001, ****P < 0.0001. One-way ANOVA with Tukey’s multiple-comparison test. See also Supplemental Figure 6.

Next, we investigated the role of FFAR1 signaling on oxidative metabolism. ATP-linked mitochondrial respiration was restrained by long-term exposure to FFAR1 agonists palmitate and GW9508 in 661W cells derived from photoreceptors (Figure 6, A and B, and Supplemental Figure 6F). Conversely, respiration was rescued by silencing *Ffar1* (Figure 6, C and D). Inhibition of fatty acid β-oxidation with etomoxir, an inhibitor of carnitine palmitoyltransferase I, further reduced ATP-linked respiration of both BSA- and palmitate-treated cells, suggesting lipids may be a source of fuel for energy production (Figure 6, A and B). However, the relative reduction in ATP-linked respiration caused by palmitate exposure was preserved despite etomoxir treatment, in line with a more global transcriptional downregulation of oxidative metabolism and TCA cycle flux (Figure 5). Conditions associated with reduced energy production resulted in increased VEGFA secretion (Supplemental Figure 6, F and G) (6) and the development of pathological neovessels in the photoreceptor layer (Figure 6E). Deleting *Ffar1*^–/–^ in *Vldlr*^–/–^ mice, despite similar circulating triglyceride levels to *Vldlr*^–/–^ mice (Supplemental Figure 6H), restored *Pgc1a*, *Ppara,* and *Sirt3* expression (Figure 4, G and H, and Supplemental Figure 6I) and prevented the formation of RAP-like neovessels (Figure 6E). More importantly, MCT-treated *Vldlr*^–/–^ mice developed more pathological neovascular RAP-like lesions, which were rescued by deleting *Ffar1* (*Vldlr*^–/–^*/Ffar1*^–/–^) (Figure 6E). Hence, FFAR1 sensed lipids and mediated the formation of RAP-like lesions in *Vldlr*^–/–^ mice.

To investigate the hypothesis that an energy deficiency drives the formation of RAP-like neovessels, we aimed to reproduce that vascular phenotype in a different transgenic model. *Sirt3*-deficient mice raised in darkness to increase photoreceptor energy demands and injected with FFAR1 agonist GW9508 also developed RAP-like lesions, whereas littermate control mice did not (Supplemental Figure 6J). Hence, a combined energy and autophagy deficit was required for RAP-like lesions to form. In short, our findings are consistent with FFAR1 sensing excess lipids and controlling fatty acid input into the TCA cycle in part via SIRT3, which restrained ATP-linked mitochondrial respiration. Energy-deficient *Vldlr*^–/–^ photoreceptors with lower α-KG levels signaled for compensatory vascular supply, albeit pathological and vision-threatening, to reinstate metabolic homeostasis.

### Enhancing autophagy rescues pathological angiogenesis.

**Figure 7:**
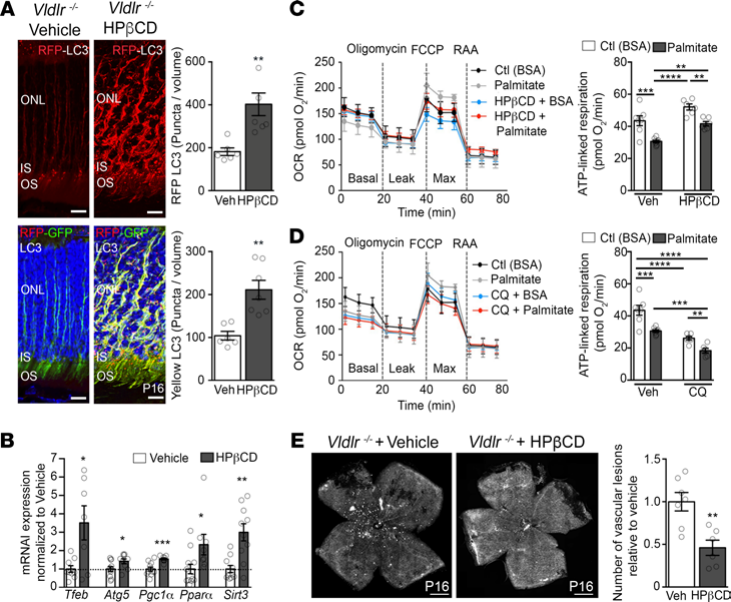
Enhancing autophagy rescues pathological angiogenesis and improves vision. (**A**) Retinal autophagy flux quantification of CAG-RFP-EGFP-LC3/*Vldlr^–/–^* mice treated with HPβCD (8 g/kg per day for 8 days; *n* = 7 retinas) compared with vehicle-treated mice (Veh; *n* = 6 retinas) at P16. ONL, outer nuclear layer; IS, photoreceptor inner segment; OS, photoreceptor outer segment. Scale bars: 10 μm. (**B**) mRNA expression in *Vldlr^–/–^* retinas of pups treated with HPβCD or vehicle. *n* = 8–12 retinas per group. (**C** and **D**) Oxygen consumption rate (OCR) and ATP-derived respiration of 661W cells treated 8 hours with palmitate or vehicle (BSA alone) in the presence or absence of drugs that raise (**C**) (HPβCD; 1 mM) or inhibit (**D**) (chloroquine, CQ; 40 μM) autophagy. *n* = 6–8 experiments per group. (**E**) Quantification of RAP-like vascular lesions (white spots) in retinal flat mounts of *Vldlr*^–/–^ mice treated with HPβCD (*n* = 6 retinas) or vehicle (Veh, *n* = 7 retinas) at P16. Scale bars:1 mm. Data are represented as mean ± SEM. *P < 0.05, **P < 0.01, ***P < 0.001, ****P < 0.0001. One-way ANOVA with Tukey’s multiple-comparison test and 2-tailed Student’s *t* test. See also Supplemental Figure 7.

Finally, we reasoned that strategies that raise autophagy might alleviate the energy shortage of photoreceptors and reduce neovascularization. We selected 2 mTOR-independent autophagy agonists: (2-hydroxypropyl)-β-cyclodextrin (HPβCD), known to increase TFEB expression (49), and trehalose (50). HPβCD is an FDA-approved drug previously shown to reduce lipid and cholesterol levels in neurons (51). Accordingly, HPβCD-treated mice had lower serum triglyceride levels (Supplemental Figure 7B), likely contributing to the ability of treated mice to regulate autophagy. As posited, HPβCD and trehalose improved autophagy flux in *Vldlr*^–/–^ retinas (Figure 7A and Supplemental Figure 7A) and increased mRNA retinal expression of *Tfeb* and *Atg5*, as well as *Pgc1a*, *Ppara,* and *Sirt3* (Figure 7B and Supplemental Figure 7C). In 661W cells, HPβCD increased TFEB transcriptional activity (Supplemental Figure 7D), raised mRNA *Sirt3* expression (Supplemental Figure 7E), and increased ATP-linked mitochondrial respiration (Figure 7C). Conversely, repressing autophagy by using chloroquine (Figure 7D) or FFAR1 agonists (palmitate and GW9508) reduced mitochondrial respiration (Figure 7, C and D, and Supplemental Figure 6F). Autophagy can therefore facilitate the production of fuel for mitochondrial respiration in photoreceptors. More importantly, autophagy agonists HPβCD and trehalose robustly reduced the number of pathological RAP-like lesions in *Vldlr*^–/–^ retinas (Figure 7E and Supplemental Figure 7, F and G), offering a potentially novel therapeutic target to alleviate neovascular retinal disease.

## Discussion

Autophagy plays an essential housekeeping role in the retina, but its contribution to neuronal energy metabolism and neovascularization and its regulation by lipids have not been described. By investigating *Vldlr*-deficient mice with high circulating lipid levels, we identified FFAR1 as a lipid sensor capable of curbing autophagy in photoreceptors associated with decreased energy production and neovascularization. FFAR1 inhibits calcineurin, which in turn prevents the translocation of transcription factor TFEB to the cell nucleus. TFEB coregulates autophagy, lysosomal biogenesis, and key enzymes of mitochondrial oxidative metabolism, including SIRT3. Energetically starved photoreceptors drive the formation of RAP-like neovascular lesions (6).

Exploring the etiology of NV-AMD is limited by the paucity of accurate animal models. *Vldlr*-deficient mice develop RAP-like lesions and, over time, AMD-like choroidal neovascularization (36). Unlike NV-AMD in humans, RAP-like lesions occur early during retinal development in mice. Although VLDLR mutations have not been directly implicated in human AMD, cone density declines in aging *Vldlr*^–/–^ retinas, as occurs in human AMD (52). We found that autophagy-related genes and *Vldlr* were enriched in photoreceptors by single-cell transcriptomics. Dysmorphic mitochondria and high levels of ROS are reported in human AMD (53) as well as in *Vldlr^–/–^* mouse retinas (52, 54) and in autophagy-deficient photoreceptors (13). We uncovered a shared metabolite signature between human RAP vitreous and *Vldlr*^–/–^ mouse retinas. Hence, despite the inherent limitations of the *Vldlr*-deficient murine RAP model, the phenotypic overlap with human RAP may help inform mechanisms driving pathological retinal neovessels.

Autophagy recycles subcellular content in part to fuel mitochondria. Photoreceptors, especially cones, have the highest autophagy flux of the retina (13). In retinal neurons, autophagy increased markedly with fasting (55), consistent with a compensatory response to diminishing nutrient supply and a role for autophagy in retinal energy production. In 661W transformed cells, derived initially from cone photoreceptors, autophagy restriction was associated with a reduction in ATP-linked respiration, and enhancing autophagy rescued mitochondrial respiration. Hence, the energy shortage that drives pathological angiogenesis toward photoreceptors in neovascular AMD might in part result from disrupted autophagy.

Lipids are energy substrates for photoreceptors in the retina (6). Increasing evidence links lipid energy metabolism and autophagy. Although this interrelationship is complex, high circulating lipid levels generally restrict autophagy in tissues with high metabolic rates (19, 40, 56). Furthermore, reduced autophagy in *Atg5*-deficient mice severely impairs lipid metabolism (19). Non-lipid nutrients like amino acids and glucose regulate autophagy in well-established fashion involving mTOR and AMPK signaling (23). However, the mechanisms linking autophagy and lipid energy metabolism are incompletely defined. FFAR1 is expressed in the retina and senses medium- and long-chain fatty acids. Lipids restrained autophagy only in the presence of FFAR1, both in retinas and in cultured 661W cells. We previously showed that FFAR1 reduces glucose uptake in the *Vldlr*^–/–^ mouse retina (6). Here, we identified FFAR1 as a coordinate transcriptional regulator of autophagy and energy metabolism.

Although lipids yield more energy per gram than glucose or proteins, the accumulation of saturated fatty acid, such as palmitate, can be toxic (57, 58). Lipotoxicity could have dire consequences in postmitotic neurons. Large concentrations of triglyceride-derived free fatty acids are released by lipoprotein lipase at the cell surface, and VLDLR facilitates cellular uptake. Inside the cells, free fatty acids can be oxidized by ROS, a necessary byproduct of oxidative phosphorylation. We believe FFAR1, which is highly expressed in the brain and retina, could transiently curtail new substrate generation by blocking autophagy and oxidative metabolism, reducing ROS production. FFAR1 was first discovered in the pancreas, where it regulates insulin secretion (24). Insulin fosters anabolic metabolism and lipid storage in adipocytes (59). In the presence of excess dietary lipids, fatty acid sensor FFAR1 might protect tissues with high metabolic rates against lipotoxicity, favoring their storage in adipose tissues (60). However, this mechanism may be maladaptive in the context of our current lipid-rich diets. Sustained exposure to higher postprandial lipids, over time, may restrain metabolic efficiency and in tissues with high metabolic requirements, such as photoreceptors, predispose to energy failure.

Common transcriptional regulation of autophagy and mitochondrial function enhances metabolic efficiency during nutrient scarcity. TFEB is considered a master regulator of lysosomal biogenesis and critical enzymes of lipid metabolism, such as PPARα and PGC1α (26). Since PGC1α binds a promoter upstream of the *Sirt3* gene (45), TFEB could indirectly regulate mitochondrial efficiency through SIRT3. SIRT3 controls the enzymatic efficiency of critical steps of fatty acid β-oxidation (47) and the TCA cycle (61), including IDH2 (48), which forms α-KG from isocitrate. Importantly, α-KG is required for the continuous degradation and maintenance of low levels of Hif1α, itself regulating VEGFA secretion and angiogenesis (62). Hence, conditions that deplete α-KG, either by substrate deficiency entering the TCA cycle or IDH2 inhibition by SIRT3, will increase VEGFA secretion, driving neovessel formation (6). Metabolite profiling confirmed the accumulation of TCA cycle metabolites upstream of IDH2 and low α-KG levels in humans with NV-AMD and in mice with RAP-like lesions. This metabolite signature correlates with increased VEGFA secretion in neovascular AMD human vitreous and mouse *Vldlr*^–/–^ retinas (6). In summary, circulating lipids restrained autophagy, retinal oxidative metabolism, and the TCA cycle in photoreceptors. In the *Vldlr*^–/–^ mouse RAP model, energy-deficient photoreceptors drove pathological neovascularization. This work may have broader implications for pathological angiogenesis in human AMD and other contexts, such as cancer, where autophagy and energy metabolism affect tumor growth (63, 64). Modulating FFAR1 may be clinically advantageous in controlling NV-AMD and other retinopathies.

## Methods

Complete methods are available in the Supplemental Methods, including a list of primer sequences and reagents (Supplemental Tables 3 and 4).

### Mice.

Mutant mice with targeted deletion of the *Vldlr* gene (*Vldlr^–/–^*), *Sirt3^–/–^*, and C57Bl/6 control mice were obtained from The Jackson Laboratory (stock 002529, 012755, and 000664, respectively). *Vldlr*^–/–^*/Ffar1*^–/–^double-mutant mice were provided in-house (6). *Vldlr^–/–^* mice were also crossed with CAG-RFP-EGFP-LC3 (The Jackson Laboratory, stock 027139) to obtain the CAG-RFP-EGFP-LC3/*Vldlr^–/–^* mouse line. All colonies were maintained and bred in standardized conditions at the CHU Sainte-Justine mice facility. Mice were exposed to identical lighting conditions, and their retinas were collected at the same time each morning. Pups weighing less than 6 grams or more than 8 grams at P14–P16 were excluded. Both littermate females and males were used.

### Humans.

All patients previously diagnosed with AMD or macular hole (without neovascularization, but requiring vitrectomy for treatment) were followed clinically and surgery was performed when indicated by standard-of-care guidelines by a single vitreoretinal surgeon. Vitreous samples were frozen on dry ice immediately after biopsy and stored at –80°C.

### Cells.

661W cells derived from cone photoreceptor (obtained from M. Al-Ubaidi, University of Oklahoma, Oklahoma City, Oklahoma, USA) (65, 66) were cultured as monolayers at 37°C, 5% CO_2_ in a humidified atmosphere in DMEM with FBS 10% supplemented with hydrocortisone (20 μg/500 mL, H-2270, Sigma-Aldrich), progesterone (20 μg/500 mL, P-8783, Sigma-Aldrich), putrescine (0.016 g/500 mL, P-7505, Sigma-Aldrich), and β-mercaptoethanol (20 μL/500 mL, M-6250, Sigma-Aldrich). No mycoplasma contamination of the cells was detected.

### Statistics.

We used 1-way ANOVA with Dunnett’s, Bonferroni’s, or Tukey’s post hoc analysis and 2-tailed Student’s *t* test (see Supplemental Table 5) to compare different groups; *P* values of less than 0.05 was considered statistically significant. The D’Agostino-Pearson or Kolmogorov-Smirnov normality tests were used to confirm a normal distribution. Data with non-Gaussian distribution were analyzed using a Mann-Whitney test (nonparametric, 2 groups). Animals were not randomized, but quantification was performed in a blinded fashion when possible. All experiments were repeated at least 3 times. Values more than 2 standard deviations from the mean were considered to be outliers and were excluded. The sample size was estimated to detect a difference of 20% with a power of 80% (1-β) and α of 0.05, in accordance with the *Guidelines for the Use of Animals in Neuroscience* (67). Results are presented as mean ± SEM.

### Data and software availability.

The Drop-Seq data reported in this paper are in NCBI’s Gene Expression Omnibus (GEO GSE110623).

### Study approval.

All animal procedures were performed in compliance with the Animal Care Committee of CHU Sainte-Justine, following the principles of the Guide for the Care and Use of Experimental Animals Id by the Canadian Council on Animal Care. The study conforms to the tenets of the Declaration of Helsinki, and approval of the human clinical protocol and informed consent were obtained from the Maisonneuve-Rosemont Hospital Ethics Committee (CER 10059).

## Author contributions

EH and JSJ conceived and designed all experiments and wrote the manuscript; EH performed most in vivo and ex vivo experiments, except for those indicated below. GC and AS performed the DROP-Seq and bioinformatics, assisted by FW and GA. JSK, AD, and NK counted RAP-like lesions and performed qPCRs. TA, NK, SP, GL, and PPR repeated and analyzed Western blots. BC, YS, AP, and LEHS generated *Vldlr*^–/–^*/Ffar1*^–/–^ mice and collected mouse samples for metabolomics and pathological lesion staining. FAR and PS collected human vitreous. KAP, CD, and CBC performed and analyzed mice and human metabolomics. SC, GAM, FAR, CBC, PS, and LEHS provided expert advice. All authors analyzed the data.

## Supplementary Material

Supplemental data

Supplemental video 1

## Version 1 02/15/2022

In-Press Preview

## Version 2 03/22/2022

Electronic publication
