# Supplementary material for: Triglyceride-derived fatty acids reduce autophagy in a model of retinal angiomatous proliferation
Source: JCI Insight. 2022 Mar 22;7(6):e154174. doi: 10.1172/jci.insight.154174 (PMC8986067; doi:10.1172/jci.insight.154174)
Supplement: Supplemental data [file jciinsight-7-154174-s218.pdf]

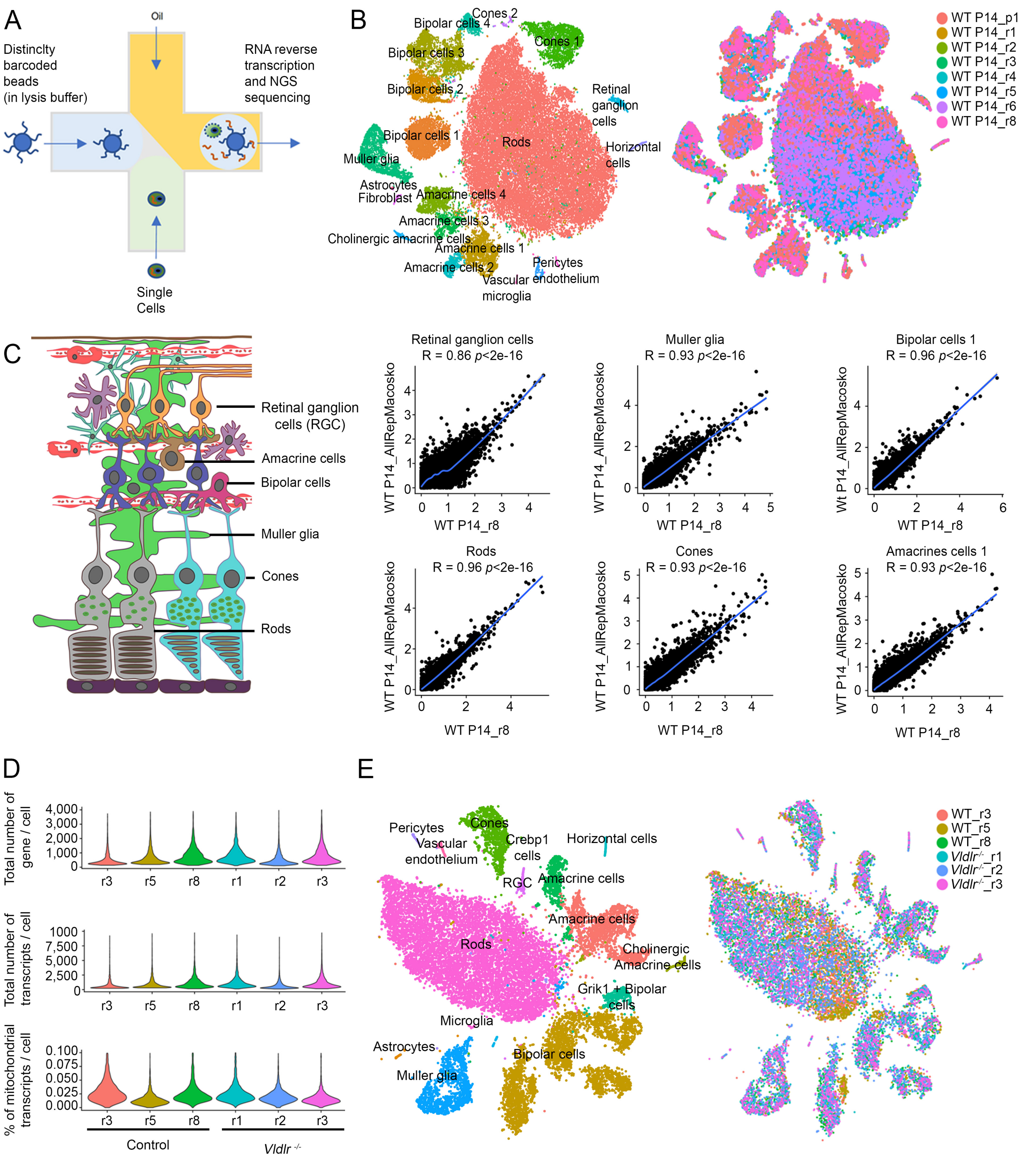

Figure S1| Quality and reproducibility of drop-seq retinal single-cell transcriptomics. (A) Schematic of single-cell mRNA sequencing method using Drop-seq, as described by Macosko et al., 2015[1]. Retinas (P14) are dissociated into individual cells, encapsulated in droplets together with microparticles (beads) coated with barcoded primers, lysed within droplets to release their mRNA, which binds to primers on the companion bead. The mRNAs are reverse-transcribed into cDNAs, and libraries are sequenced on NextSeq 500. (B) Annotated dimensionality reduction (tSNE) plots showing overlapping cell clusters of P14 WT control retinas obtained from public data (p1 and r1 to r6)[1] and our own replicate (r8). n = 8 retinas. (C) Schematic diagram of retinal cell types (left panel) and transcriptomic correlation in most prominent cell types across replicates from P14 WT control retinas obtained from public data (right panel)[1], and our own replicate (r8). (D) Total number of genes (nGene), the unique molecular identifiers (UMIs), and the ratio of mitochondrial genes (percent.mito, as percentage of nGene) detected in each barcoded cell from WT and *Vldlr*$^{-/-}$ retina replicates. n = 3 per condition. (E) Annotated dimensionality reduction (tSNE) plots (left) showing overlapping (right) cell clusters from P14 WT and *Vldlr*$^{-/-}$ retinas. n = 3 per condition.

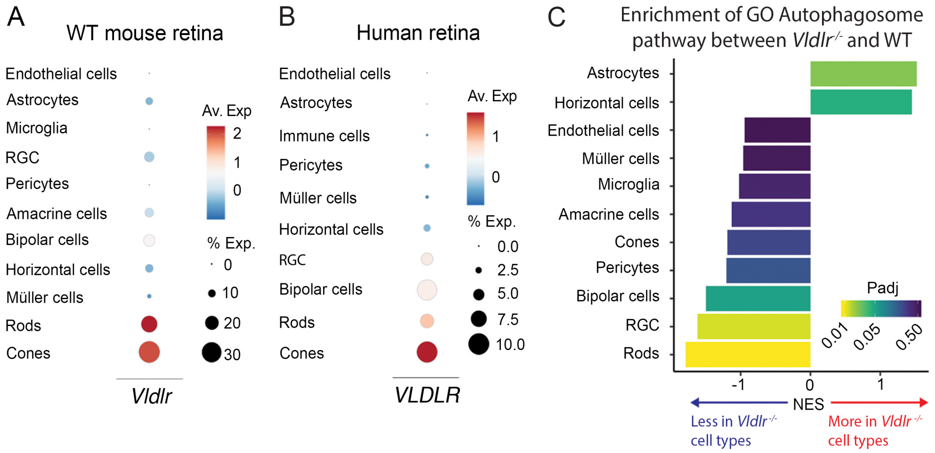

Figure S2| Single-cell RNAseq analysis of retinal *Vldlr* expression and the impact of its deletion on autophagy. (A) Dot plot of *Vldlr* transcript expression in WT retinal cell types from P14 mice. (B) Dot plot of *VLDLR* transcript expression in adult retinal cell types from human donors (public single-cell RNAseq data obtained from GEO: GSE148077). (C) Normalized Enrichment Scores (NES) for the GO_AUTOPHAGOSOME gene set from GSEA of differentially expressed genes between *Vldlr*$^{-/-}$ and WT across retinal cell types. n = 3801 *Wt* and 5642 *Vldlr*$^{-/-}$ cells pooled from 3 retinas per group.

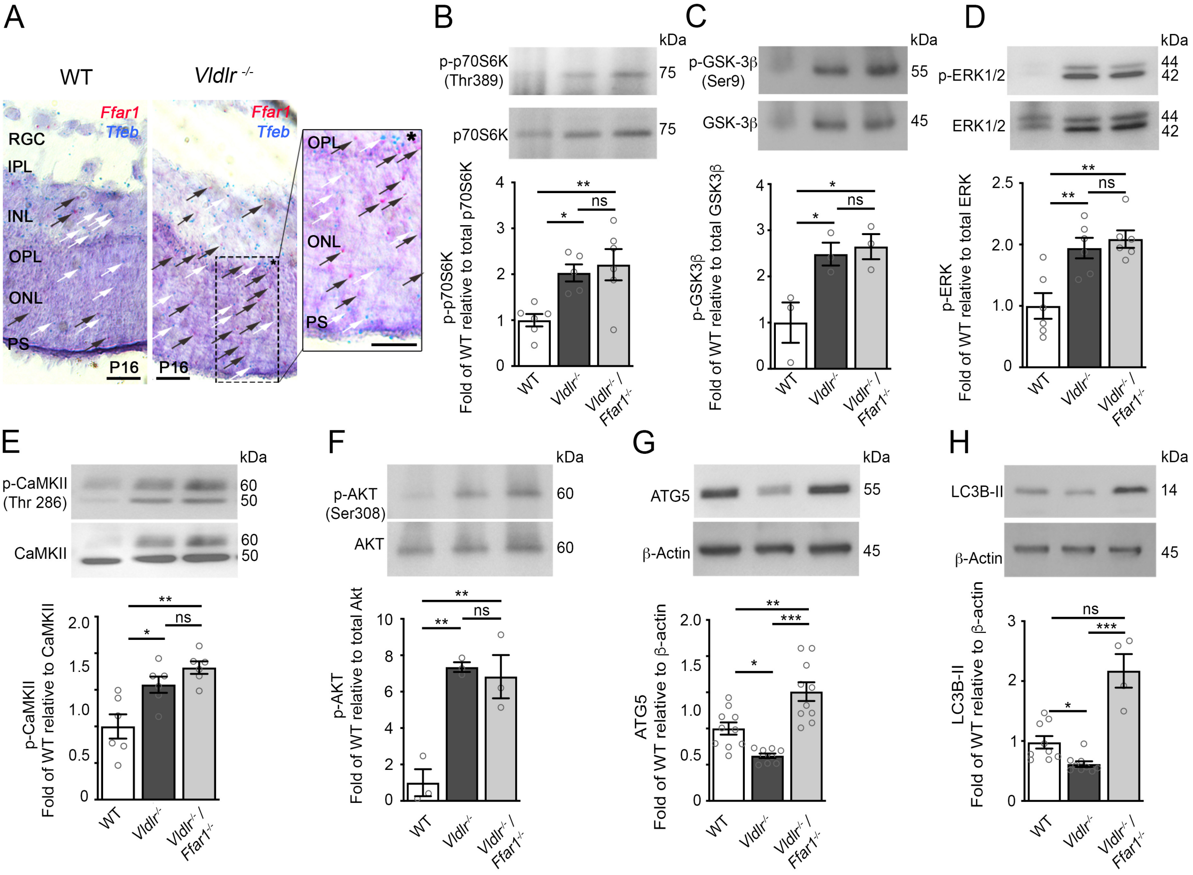

Figure S3 | Free fatty acid receptor 1 supresses TFEB. (A) Localization of retinal *Tfeb* (blue dots, white arrows) and *Ffar1* (red dots, black arrows) by RNAscope *in situ* hybridization. PS: photoreceptor posterior segment, ONL: Outer Nuclear Layer, OPL: Outer Plexiform Layer, INL: Inner Nuclear Layer, IPL: Inner Plexiform Layer, RGC: Retinal Ganglion Cells. Scale bars: 50 μm and 25 μm. (B-F) Western blot (WB) quantifications of known TFEB regulators and their phosphorylation status (p70S6K, GSK3β, ERK1/2, CaMKII, AKT) in WT, *Vldlr*$^{-/-}$ and *Vldlr*$^{-/-}$ / *Ffar1*$^{-/-}$ retinas at P16. None of these pathways were rescued by *Ffar1* deletion in *Vldlr*$^{-/-}$ retinas. n = 6-12 retinas per group. (G-H) WB quantifications of ATG5 and LC3B-II in WT, *Vldlr*$^{-/-}$, and *Vldlr*$^{-/-}$ / *Ffar1*$^{-/-}$ retinas at P16. n = 8-20 retinas per group. Data are represented as mean ± SEM. ns: not significant, * P <0.05, ** P < 0.01, *** P < 0.001. One-way ANOVA with Tukey's and Dunn's multiple comparisons test.

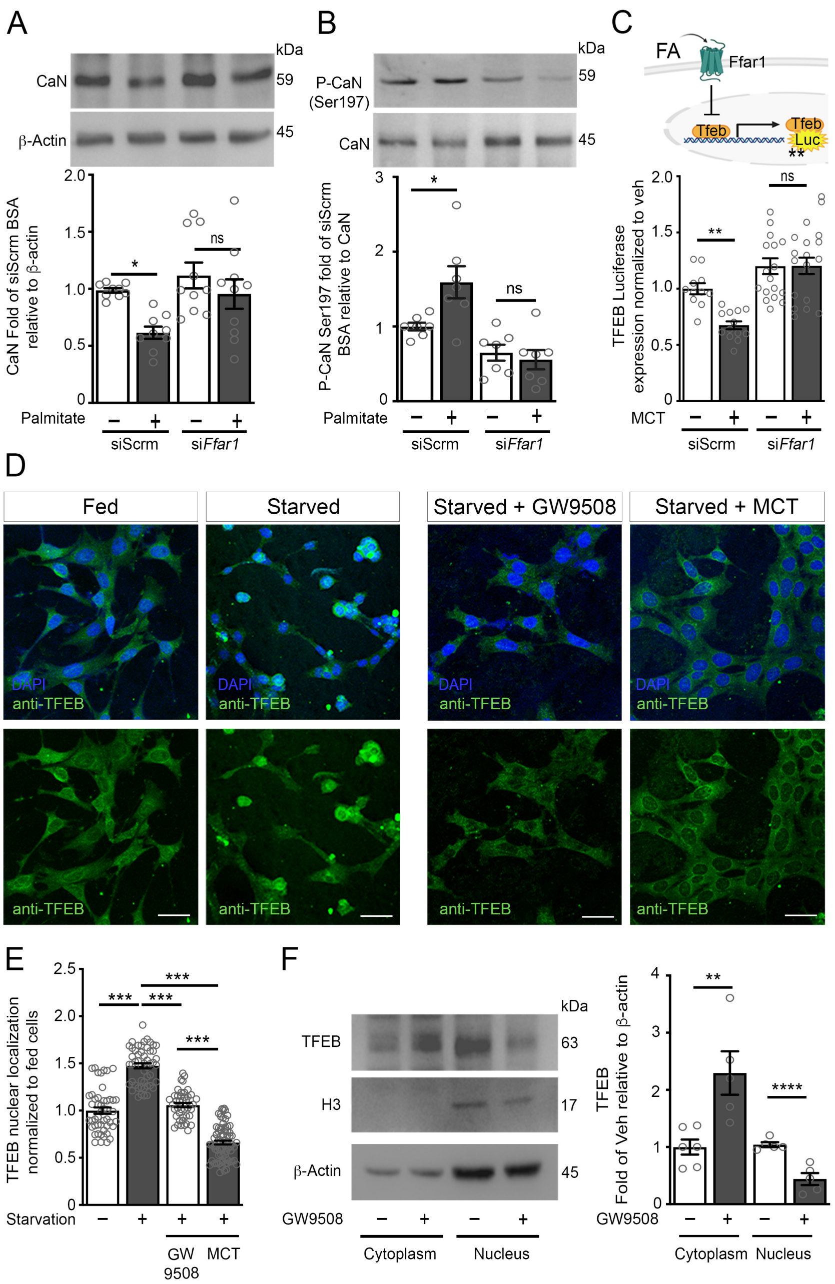

Figure S4| FFAR1 suppresses trancription factor EB. (A,B) Western blot quantifications of calcineurin phosphorylation in 661W cells silenced or not for *Ffar1* (siRNA), and treated with its agonist, palmitate, or corresponding vehicle. n = 7-10 independent experiments. (C) Schematic of luciferase reporter assay (top) and its quantifications (bottom). Stable 661W cell transfections of a vector containing the TFEB promoter driving luciferase expression; *Ffar1* was silenced (siRNA) or not in these cells treated with MCT (0.4%, 17h) or vehicle. Luc: Luciferase. n = 11-18 experiments. (D,E) Representative immunofluorescence of TFEB nuclear translocation in fed or starved 661W cells. Starved cells were also treated with FFAR1 agonist, GW9508 or MCT. Scale bar: 25 μm. n = 43-76 cells per group. (F) Western blots of TFEB protein expression in nuclear and cytoplasmic 661W cell fractions, treated or not with GW9508 (30 μM, 17h). n = 5-6 per group. Data are presented as mean ± SEM. * $P < 0.05$, ** $P < 0.01$, *** $P < 0.001$, **** $P < 0.0001$. One-way ANOVA with Bonferroni's comparisons and two-tailed Student's t-test.

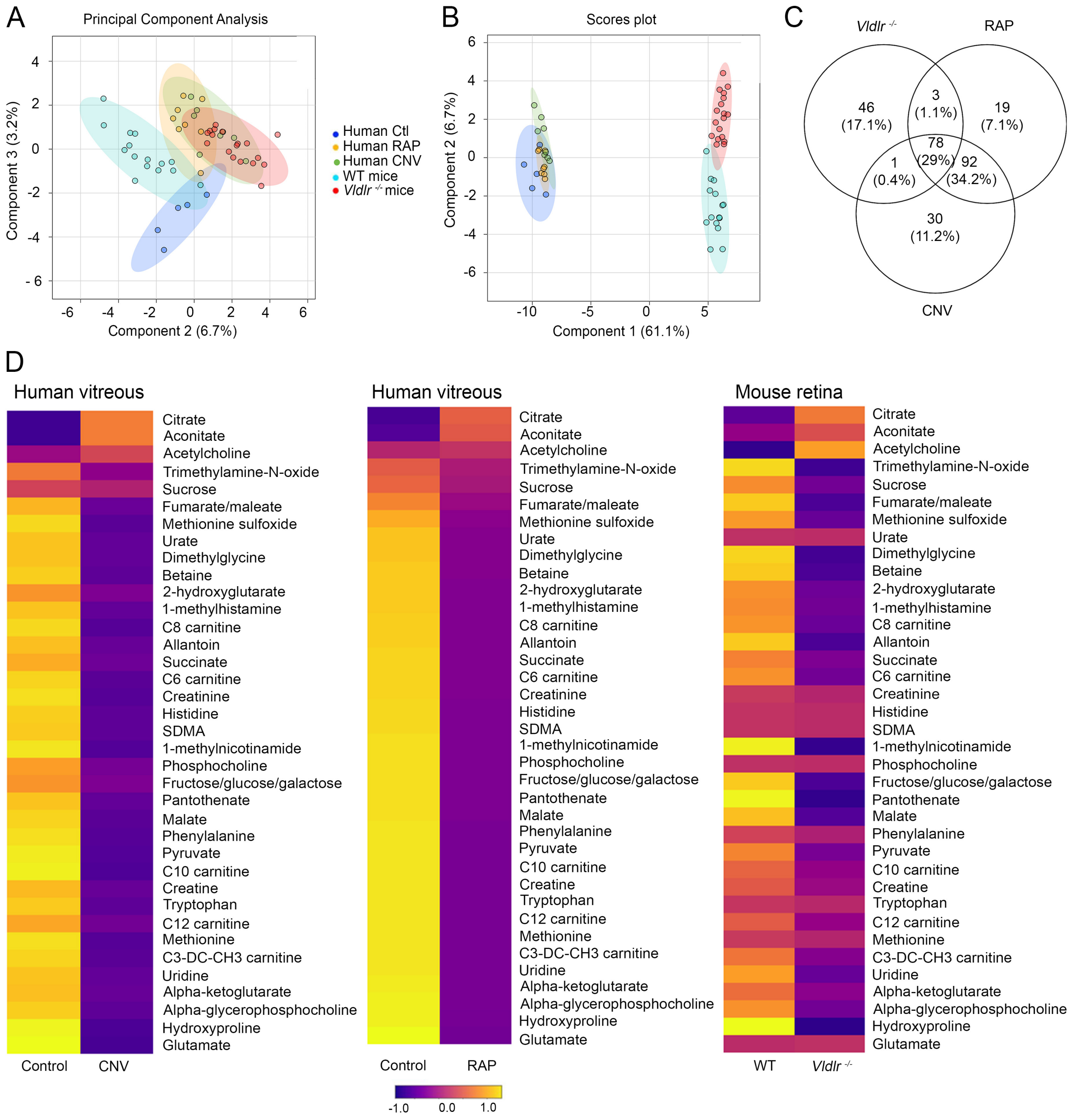

Figure S5| Human RAP vitreous and *Vldlr$^{-/-}$* RAP-like retinas share metabolite signatures. (A,B) Principal component (PC) analysis of metabolomics profiles from human RAP and CNV vitreous, and *Vldlr$^{-/-}$* mouse retinas compared to their respective control. (A) PC2 and PC3 segregate healthy and diseased RAP samples from humans and mice, which share common metabolite signatures, (B) whereas PC1 is strongly species-dependent. n = 8 RAP, 7 CNV and 5 human control (macular hole) vitreous, compared to 18 *Vldlr$^{-/-}$* and 15 WT mouse retinas. (C) Venn diagram of annotated metabolites detected in human RAP vitreous (223 metabolites) and mice *Vldlr$^{-/-}$* retina (128 metabolites); 82 (30.4%) metabolites were shared by both. (D) Heat map of the shared metabolites between human control (macular holes, n = 5) and RAP vitreous (n = 8 samples) and CNV vitreous (n=7 samples), which correlates with WT (n = 15) and *Vldlr$^{-/-}$* (n = 18) mouse retinas.

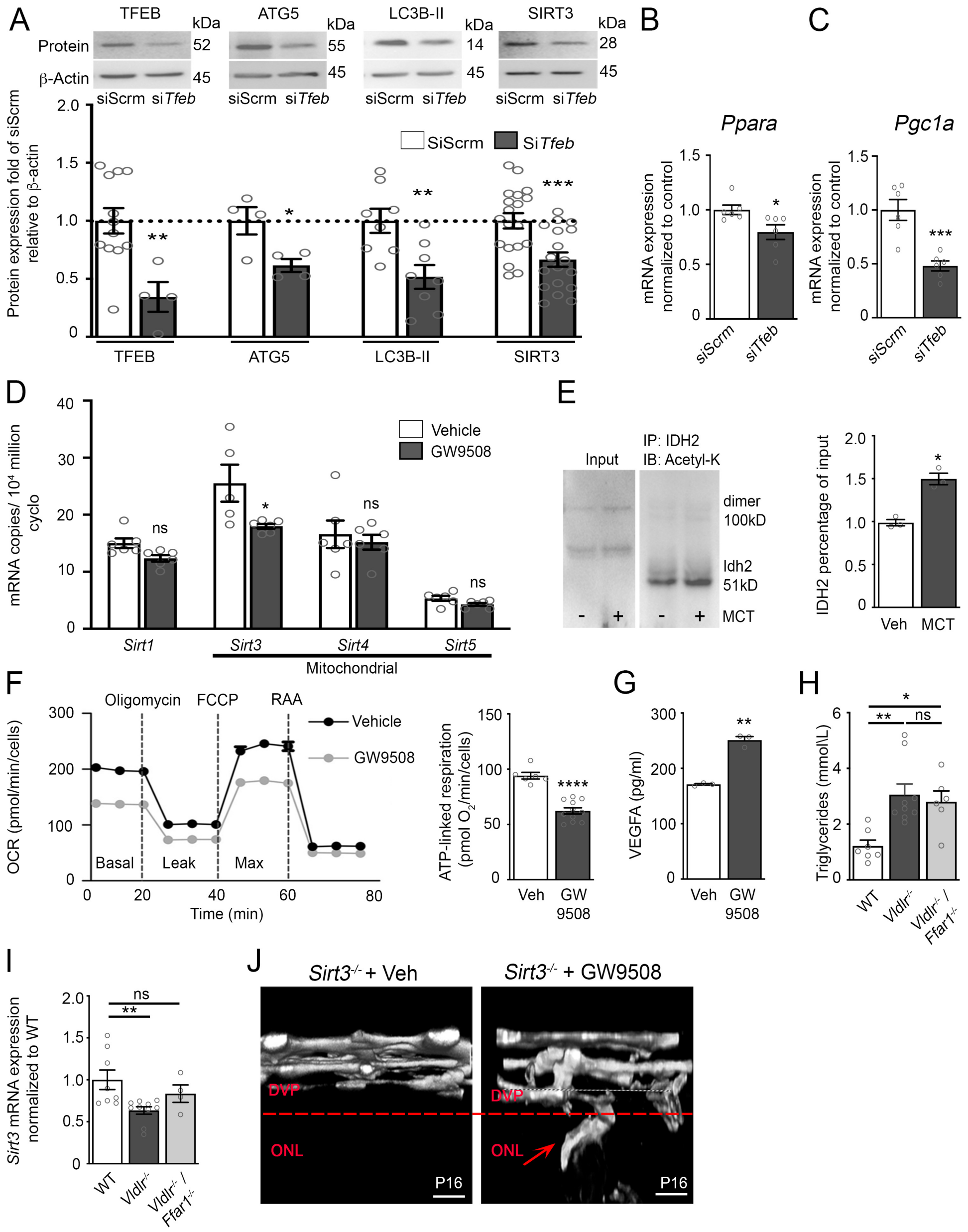

Figure S6 | FFAR1 restrains oxidative metabolism via Sirtuin-3. (A) Western blot quantifications of TFEB, ATG5, LC3B-II and SIRT3 protein expression in *Tfeb*-depleted (siRNA) 661W cells compared to control (scrambled siRNA, siScrm). n = 4-19 experiments per group. (B-C) *Ppara* and *Pgc1a* mRNA expression in 661W cells transfected with the siRNA of TFEB (65ng). n = 3 independent experiments per condition. (D) *Sirt1* and mitochondrial sirtuins (*Sirt3, Sirt4* and *Sirt5*) mRNA expression in 661W photoreceptor cells, treated or not with FFAR1 agonist GW9508 (14μM, 17h). n = 3 independent experiments per condition. (E) Immunoprecipitation (IP) of isocitrate dehydrogenase-2 (IDH2) in 661W cells treated with MCT or vehicle (Veh). K: Lysine acetylation residues; IB: immunoblotting. n = 3 independent experiments. (F) Oxygen consumption rate (OCR) and ATP production of 661W cells treated with FFAR1 agonist, GW9508 (14 μM, 8h) or vehicle. n = 6-11 experiments per group. (G) ELISA of VEGFA secretion of 661W photoreceptors treated or not with GW9508 agonist (14 μM, 6 h) or vehicle. n = 3 independent experiments. (H) Serum triglyceride concentrations of P16 WT, *Vldlr*$^{-/-}$ and *Vldlr*$^{-/-}$/ *Ffar1*$^{-/-}$ mice (n = 7, 10, and 6, respectively). (I) *Sirt3* mRNA expression of P16 WT (n = 16), *Vldlr*$^{-/-}$ (n = 29) and *Vldlr*$^{-/-}$/ *Ffar1*$^{-/-}$ (n = 8) retinas. (J) Representative 3D confocal images and pathological lesions (red arrow) in *Sirt3*$^{-/-}$ mice treated with FFAR1 agonist (GW9508, 14 μM, twice a day) or vehicle. DVP: deep vascular plexus, ONL: outer nuclear layer. Scale bars: 200 μm. Data are represented as mean ± SEM. * P <0.05, ** P < 0.01, *** P < 0.001, **** P < 0.0001. Two-tailed Student's t-test and One-way ANOVA with Bonferroni's comparisons.

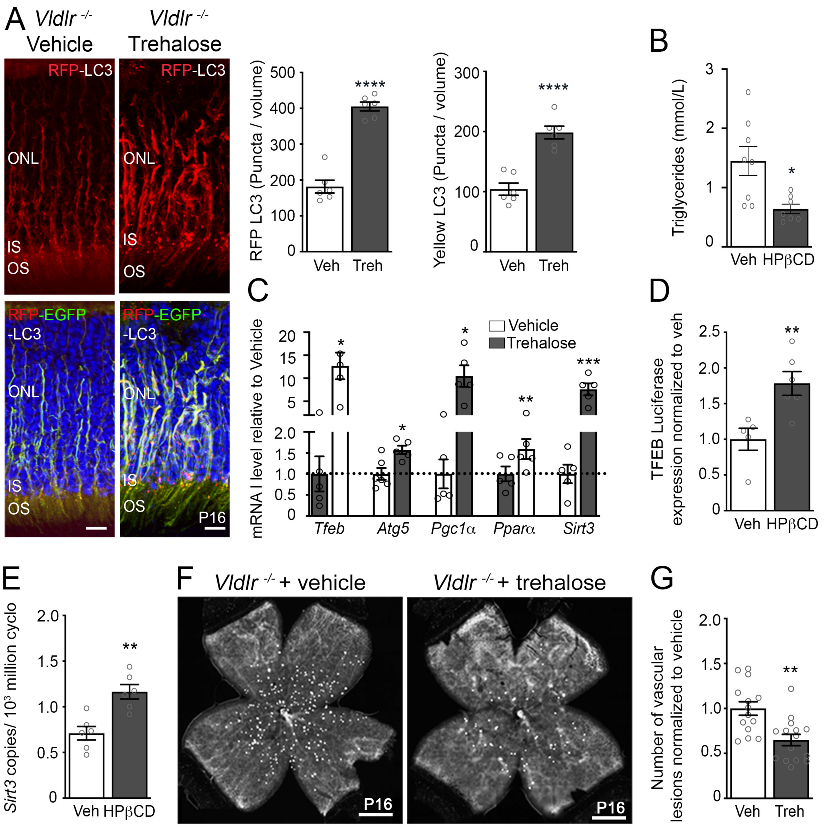

Figure S7 | Enhancing autophagy rescues pathological angiogenesis. (A) Retinal autophagy flux quantification of CAG-RFP-EGFP-LC3 / *Vldlr*$^{-/-}$ mice treated with trehalose (Treh, 2g/kg, 8days) or vehicle, at P16. Control eye results from multiple experiments performed simultaneously with Figure 6A. n = 6 retinas per conditions. Scale bars: 10 μm. (B) Serum triglyceride concentrations of P16 *Vldlr*$^{-/-}$ mice treated or not with HPβCD (n = 7-8) (C) mRNA expression in *Vldlr*$^{-/-}$ retinas of pups treated with trehalose or vehicle at P16. n = 10-12 retinas per group. (D,E) TFEB and *Sirt3* expression in 661W photoreceptor cells treated with HPβCD or vehicle, using the (D) luciferase reporter assay and (E) qRT-PCR. n = 5-6 independent experiments per conditions. (F,G) Quantification of RAP-like vascular lesions (white dots) in *Vldlr*$^{-/-}$ mice at P16 treated with trehalose (Treh) or vehicle. n = 14 retinas (vehicle) and 15 retinas (trehalose). Scale bars: 1mm. Data are represented as mean ± SEM. * P <0.05, ** P < 0.01, *** P < 0.001, **** P < 0.0001. Two-tailed Student's t-test and One-way ANOVA with Tukey's and Dunn's multiple comparisons test.

SUPPLEMENTAL FIGURE LEGENDS

Figure S1. Quality and reproducibility of drop-seq retinal single-cell transcriptomics. Related to Figure 1

(**A**) Schematic of single-cell mRNA sequencing method using Drop-seq, as described by Mascosko et al, 2015. Mouse retinas (P14) are dissociated into individual cells, encapsulated in droplets together with microparticles (beads) coated with barcoded primers, lysed within droplets to release their mRNA, which then binds to primers of their companion bead. The mRNAs are reverse-transcribed into cDNAs and libraries are sequenced on NextSeq 500.

(**B**) Annotated dimensionality reduction (tSNE) plots showing the overlapping of cell clusters of P14 WT control retinas obtained from public data (p1 and r1 to r6, Mascosko et al, 2015) and our own replicate (r8). n = 8 retinas.

(**C**) Schematic diagram of retinal cell types (left panel) and transcriptomic correlation in most prominent cell types across replicates from P14 WT control retinas obtained from public data (right panel, Mascosko et al, 2015) and our own replicate (r8). n = 8 retinas

(**D**) Total number of genes (nGene), the total number of unique molecular identifiers (UMIs), and the ratio of mitochondrial genes (percent.mito, as percentage of nGene) detected in each barcoded cell from WT and *Vldlr*$^{-/-}$ retina replicates. n = 3 retinas per condition.

(**E**) Annotated dimensionality reduction (tSNE) plots (left) showing the overlapping (right) of cell clusters from P14 WT and *Vldlr*$^{-/-}$ retina replicates. n = 3 retinas per condition.

Figure S2. Single-cell RNAseq analysis of retinal *Vldlr* expression and the impact of its deletion on autophagy.

Related to Figure 1

(**A**) Dot plot of *Vldlr* transcript expression in WT retinal cell types from P14 mice.

(**B**) Dot plot of *VLDLR* transcript expression in adult retinal cell types from human donors (public single-cell RNAseq data obtained from GEO: GSE148077).

(**C**) Normalized Enrichment Scores (NES) for the GO_AUTOPHAGOSOME gene set from GSEA of differentially expressed genes between *Vldlr*$^{-/-}$ and WT across retinal cell types.

n = 3801 WT and 5642 *Vldlr*$^{-/-}$ cells pooled from 3 retinas per group.

Figure S3. Free fatty acid receptor 1 suppresses TFEB

Related to Figure 3

(A) Localization of retinal *Tfeb* (blue dots, white arrows) and *Ffar1* (red dots; black arrows) by RNAscope *in situ* hybridization, at P16. PS: photoreceptor posterior segment, ONL: Outer Nuclear Layer, OPL: Outer Plexiform Layer, INL: Inner Nuclear Layer, IPL: Inner Plexiform Layer, RGC: Retinal Ganglion Cells. Scale bars: 50 and 25μm.

(**B-F**) Western blot (WB) quantifications of known TFEB regulators and their phosphorylation status (p70S6K, GSK3β, ERK1/2, CaMKII and AKT) in WT, *Vldlr*$^{-/-}$*, and Vldlr*$^{-/-}$*/ Ffar1*$^{-/-}$ at P16. None of these pathways were rescued by Ffar1 deletion in *Vldlr*$^{-/-}$ retinas. n = 6-12 retinas per group. (**G-H**) WB quantifications of ATG5 and LC3B-II in WT, *Vldlr*$^{-/-}$ and *Vldlr*$^{-/-}$*/ Ffar1*$^{-/-}$ retinas at P16. n = 8-20 retinas per group.

Data are represented as mean ± SEM. ns: not significant, * $p <0.05$, ** $p < 0.01$, *** $p < 0.001$. One-way ANOVA with Tukey's and Dunn's multiple comparisons test.

Figure S4. **FFAR1 Suppresses transcription factor EB**

Related to Figure 3

(**A-B**) Western blot quantifications of calcineurin phosphorylation in 661W cells silenced or not for *Ffar1* (siRNA), and treated with its agonist, palmitate, or corresponding vehicle. n = 7-10 independent experiments.

(**C**) Schematic of luciferase reporter assay (top) and its quantifications (bottom). Stable 661W cell transfections of a vector containing the TFEB promoter driving luciferase expression; Ffar1 was silenced (siRNA) or not in these cells treated with MCT (0.4%, 17h) or vehicle. Luc: Luciferase. n = 11-18 experiments.

(**D-E**) Representative immunofluorescence of TFEB nuclear translocation in fed or starved 661W cells. Starved cells were also treated with FFAR1 agonist, GW9508 or MCT. Scale bar: 25 μm. n = 43-76 cells per group.

(**F**) Western blots of TFEB protein expression in nuclear and cytoplasmic 661W cell fractions, treated or not with GW9508 (30μM, 17h). n = 5-6 experiments per group.

Data are represented as mean ± SEM. * $p <0.05$, ** $p < 0.01$, *** $p < 0.001$, **** $p < 0.0001$. One-way ANOVA with Bonferroni's comparisons and two-tailed Student's t-test.

Figure S5. Human RAP vitreous and *Vldlr*$^{-/-}$ RAP-like retinas share metabolite signatures
Related to Figure 4

(**A-B**) Principal component (PC) analysis of metabolomics profiles from human RAP and CNV vitreous, and mouse *Vldlr*$^{-/-}$ retinas compared to their respective control. (**A**) PC2 and PC3 segregate healthy and diseased RAP samples from humans and mice, which share common metabolite signatures, (**B**) whereas PC1 is strongly species-dependent. n = 8 RAP, 7 CNV, 5 human control (macular hole) vitreous, compared to 18 *Vldlr*$^{-/-}$ and 15 WT retinas.

(**C**) Venn diagram of annotated metabolites detected in human RAP vitreous (192 metabolites) and mice *Vldlr*$^{-/-}$ retina (129 metabolites); 83 (34.9%) metabolites were shared by both.

(**D**) Heat map of the shared metabolites between human control (macular holes, n = 5) and RAP vitreous (n = 8 samples), which correlates with mice WT (n = 15) and *Vldlr*$^{-/-}$ (n = 18) retinas.

Figure S6. FFAR1 restrains oxidative metabolism via sirtuin-3
Related to Figure 5

(**A**) Western blot quantifications of TFEB, ATG5, LC3B-II and SIRT3 protein expression in *Tfeb*-depleted (siRNA) 661W cells compared to control (scrambled siRNA, siScrm). n = 4-19 experiments per group.

(**B-C**) *Ppar*α and *Pgc1*α mRNA expression in 661W cells transfected with the siRNA of TFEB (65ng). n = 3 independent experiments per condition.

(**D**) *Sirt1* and mitochondrial sirtuins (*Sirt3*, *Sirt4* and *Sirt5*) mRNA expression in 661W photoreceptor cells, treated or not with FFAR1 agonist GW9508 (14μM, 17h). n = 3 independent experiments per condition.

(**E**) Immunoprecipitation (IP) of isocitrate dehydrogenase-2 (IDH2) in 661W cells treated with MCT or vehicle (Veh). K: Lysine acetylation residues; IB: immunoblotting. n = 3 independent experiments.

(**F**) Oxygen consumption rate (OCR) and ATP production of 661W cells treated with FFAR1 agonist, GW9508 (14 μM, 8h) or vehicle. n = 6-11 experiments per group.

(**G**) ELISA of VEGFA secretion of 661W photoreceptors treated or not with GW9508 agonist (14 μM, 6 h) or vehicle. n = 3 independent experiments.

(**H**) Serum triglyceride concentrations of P16 WT, *Vldlr*$^{-/-}$ and *Vldlr*$^{-/-}$ / *Ffar1*$^{-/-}$ mice (n = 7, 10, and 6, respectively).

(**I**) *Sirt3* mRNA expression of P16 WT (n = 16), *Vldlr*$^{-/-}$ (n = 29) and *Vldlr*$^{-/-}$ / *Ffar1*$^{-/-}$ (n = 8) retinas.

(**J**) Representative 3D confocal images and pathological lesions (red arrow) in *Sirt3*$^{-/-}$ mice treated with FFAR1 agonist (GW9508, 14 µM, twice a day) or vehicle. DVP: deep vascular plexus, ONL: outer nuclear layer. Scale bars: 200 mm. Data are represented as mean ± SEM. * P <0.05, ** P < 0.01, *** P < 0.001, **** P < 0.0001. Two-tailed Student's t-test and One-way ANOVA with Bonferroni's comparisons.

Figure S7. Enhancing autophagy rescues pathological angiogenesis

Related to Figure 6

(**A**) Retinal Autophagic flux quantification of CAG-RFP-EGFP-LC3/*Vldlr*$^{-/-}$ mice treated with trehalose (Treh, 2g/kg, 8days) or vehicle, at P16. Control eyes results from multiple experiments which were performed simultaneously with Figure 6A. n = 6 retinas per conditions. Scale bars: 10µm.

(**B**) Serum triglyceride concentrations of P16 *Vldlr*$^{-/-}$ mice treated or not with HPβCD (n = 7-8)

(**C**) mRNA expression in *Vldlr*$^{-/-}$ retinas of pups treated with trehalose or vehicle, at P16. n = 10-12 retinas per group.

(**D-E**) TFEB and *Sirt3* expression in 661W photoreceptor cells treated with HPβCD or vehicle, using the (**D**) luciferase reporter assay and (**E**) qRT-PCR. n = 5-6 independent experiments per conditions.

(**F-G**) Quantification of RAP-like vascular lesions (white dots) at P16 in *Vldlr*$^{-/-}$ mice treated with trehalose (Treh) or vehicle. n = 14 retinas (vehicle) and 15 retinas (trehalose). Scale bars: 1mm. Data are represented as mean ± SEM. * p <0.05, ** p < 0.01, *** p < 0.001, **** p < 0.0001. Two-tailed Student's t-test and One-way ANOVA with Tukey's and Dunn's multiple comparisons test.

Supplemental table 1. Differentially expressed genes between Vldlr-deficient and wild-type photoreceptors from P14 mouse retina Vldlr-deficient and wild-type photoreceptors from P14 mouse retina.
n = 1219 WT and 1341 *Vldlr*$^{-/-}$ cells pooled from 3 retinas per group, P14.

50 most Up regulated genes when comparing Vldlr to WT

Genes	Avg Log Fold Change	*P*_val_adj
EIF2S3Y	0.80759449	3.13E-25
ARR3	0.803543457	1.68E-12
OPN1MW	0.738820236	9.48E-09
GM26924	0.733428803	2.93E-30
JUN	0.711231388	1.47E-08
GNAT2	0.661227468	0.000000295
OPN1SW	0.633983047	0.000056
EGR1	0.631045262	1.06E-09
SRP54B	0.628923641	3.65E-17
MT-CO1	0.61409481	1.68E-39
RPS28	0.585583228	5.13E-15
KCNE2	0.570558837	0.000259488
4930447C04RIK	0.563350146	0.000000076
TSHZ2	0.562631508	3.94E-09
MT-ND5	0.561259738	1.6E-50
TRPM1	0.556454959	0.002542814
CNGB3	0.547807716	0.0000123
GNB3	0.54529131	3.06E-08
CHGB	0.538484429	1.14E-18
GABRB3	0.528745465	0.00000385
CAMK2B	0.518656348	0.0000169
LRTM1	0.517631365	0.000355749
AGR2	0.51193337	0.00000172
GNGT2	0.511920483	0.00000441
KCNMA1	0.509610184	0.00000807
DDX3Y	0.508595113	3.04E-09
SCG3	0.498744796	0.00000498
BC048943	0.496906533	0.008204901
TMSB10	0.493563046	0.00000977
UACA	0.492759473	0.000000155
CLTB	0.478511118	0.000011
FAM19A3	0.478483588	0.00000099
NR4A1	0.474491695	0.0000136
SPHKAP	0.468067375	0.0000542
A930033H14RIK	0.465143232	0.0000596
RPL18A	0.463218911	0.00000406
SLC38A1	0.454916859	0.000395071
ECE1	0.451506309	0.000592119
NAV1	0.451113167	0.003968215
MT-ND6	0.450214551	0.000000787
GM10036	0.447984982	1.91E-16
IMPG1	0.4475081	2.84E-10
NRCAM	0.444473069	0.001167809
AKAP11	0.442974692	0.000000705
ELOVL6	0.433409081	0.002660639
RAPGEF5	0.430483858	0.013447277
MAP2	0.429922174	4.38E-18
SPTSSA	0.42880655	0.003022442
RWDD4A	0.426434664	0.0000829
EIF1A	0.425306415	0.01109794

50 most Down regulated genes when comparing Vldlr to WT

Genes	Avg Log Fold Change	*P*_val_adj
XIST	-2.607412479	1.34E-224
GM10073	-1.050068859	1.92E-66
GUCA1B	-0.984049247	1.49E-81
GM4204	-0.968958973	1.08E-62
RPSA-PS10	-0.962978098	9.45E-59
WDR89	-0.892159822	3.68E-51
GM10288	-0.884376168	1.45E-53
UNC119	-0.869195726	2.63E-82
ANKRD12	-0.824787077	3.96E-58
PCLO	-0.822798676	4.34E-41
CASZ1	-0.769421365	3.03E-51
SCAND1	-0.722541017	6.86E-38
NDUFS2	-0.721296637	1.42E-42
CITED2	-0.718308825	1.08E-35
RPL9-PS6	-0.702992876	8.99E-38
ARL4D	-0.675897534	1.18E-30
TSIX	-0.654450079	9.33E-30
PTMS	-0.652853131	9.84E-37
SLCO4A1	-0.65090563	7.5E-30
A430035B10RIK	-0.647352621	1.36E-28
C2CD2L	-0.642410047	3.59E-30
RPS10-PS1	-0.63763543	1.3E-32
LAMP1	-0.633312389	7.64E-30
EIF3J2	-0.630660927	1E-28
YTHDC1	-0.611639984	7.28E-26
RAX	-0.610116337	1.67E-25
PINK1	-0.609103352	1.12E-27
RPL30	-0.607780136	4.85E-27
NRL	-0.59460851	2.65E-53
VAX2OS	-0.588020023	5.83E-29
ZFP91	-0.58039594	5.61E-22
DRD4	-0.564508156	7.67E-29
EIF3F	-0.561275482	2.53E-24
ARGLU1	-0.555389349	6.87E-20
RNF220	-0.551483227	8.07E-25
SET	-0.536781189	1.63E-20
B020018G12RIK	-0.534784722	4.37E-21
SPRY2	-0.528980583	4.19E-20
GM4707	-0.520322103	2.57E-26
TUBA1B	-0.519432745	5.25E-22
DDB1	-0.516980242	1.79E-19
YBX3	-0.514849603	7.37E-33
PTOV1	-0.514287899	2.32E-21
BTG1	-0.502889028	2.97E-17
GM10250	-0.502571011	1.05E-18
EIF5B	-0.501863699	5.08E-21
JUND	-0.500521459	2.04E-18
YBX1	-0.500349096	2.52E-19
RPL3	-0.496910273	1.73E-23
CAMK2N1	-0.494958103	9.98E-17

Table S2. Human Clinical Information

Human Vitreous	Gender	Age	Eye collected	OCT Thickness μm	Disease status	Past Medical History
Macular Hole (MH)	F	81	OD	-	Control	-
	M	67	OD	-	Control	-
	F	74	OD	-	Control	Diabetes type 2
	M	65	OS	-	Control	Hypertension, Dyslipidemia
	M	75	OD	-	Control (MH) & Cataract	Coronary artery disease
RAP	M	80	OD	418	Inactive	Hypertension, Diabetes type 2
	F	91	OS	339	Active	
	F	85	OD	382	Active	Osteoporosis, Hypertension
	F	94	OS	1077	Naive	Hypertension
	F	82	OD	244	Inactive	Hypertension, Dyslipidemia
	F	84	OD	221	Inactive	Osteoporosis, Hypertension, Anemia
	F	80	OS	321	Inactive	-
	F	87	OD	-	Naive	-
	F	91	OS	297	Active	Hypertension
CNV	F	96	OS	1150	Naive	Coronary artery disease, Stroke, Breast cancer, Dyslipidemia
	F	74	OD	289	Naive	Hypertension
	F	90	OD	231	Inactive	Hypertension, Dyslipidemia, Diabetes type 2, Hypothyroidism, B12 deficiency
	F	75	OD	361	Naive	Osteoporosis, Dyslipidemia, Hypertension
	F	79	OD	239	Inactive	-
	F	86	OS	168	Naive	Hypertension, Osteoporosis
	F	75	OD	312	Inactive	Dyslipidemia, Hypothyroidism
	F	85	OD	250	Naive	Hypertension, Dyslipidemia, Coronary artery disease, Hypothyroidism
	F	82	OD	-	Inactive	-
	F	79	OD	-	Inactive	-
	F	96	OS	1150	Naive	Coronary artery disease, Stroke, Breast cancer, Dyslipidemia

Supplementary Table 2. Clinical information of human subjects. Vitreous was collected either in the right (OD) or left (OS) eye of patients with macular hole (control), retinal angiomatous proliferation (RAP) and choroidal neovascularization (CNV). All patients with active neovascular disease were treated with Bevacizumab (1.25mm/0.05ml) after vitreous collection. Naïve neovascular disease refers to patients that had not received prior anti-VEGF injections. Active disease characterizes patients that developed new neovascular lesions despite prior anti-VEGF treatment. Inactive disease defines subjects with stable neovascular retinal lesions after anti-VEGF treatment.

Table S3. Primer Sequences

Gene	Forward (5' – 3')	Reverse (5' – 3')	Source
Atg 5	CGCCCCTGAAGATGGAGAGA	AGAGGGGTTTCCAGCATTGG	This paper
Atg 12	TAAACTGGTGGCCTCGGAAC	CCATCACTGCCAAAACACTCA	This paper
Atp6V0a1	CTT TGG TGG GAT CAG GGT GG	AGT CAA ACT CTT CTG CGT CC	Settembre et al, 2013
Atp6V0b	TGT GGT CGA ATC TGG GCA TT	CCA CCC CCA ATG ATG GAA GAA	Settembre et al, 2013
Atp6V0c	GGG ATC ATC GCC ATC TAC GG	ACC CAG TTG AAG AAA ACT CCT GT	Settembre et al, 2013
Atp6V0e1	GGG TCC TAA CCG GGG AGT TAT	TTG AGC TGT GCC AGA ATT GC	Settembre et al, 2013
Atp6V1a	ATG CCC TTT TCC CGT GTG TT	CCG CAG CCG ACA TAG ATG AT	Settembre et al, 2013
Atp6V1b2	AAT GTG CTG CCC TCA CTC TC	GCA TAG CAC GCA TAC AAC TGG	Settembre et al, 2013
Atp6V1c1	CCT AAA GTC TCG GGC GTC TG	TAG CAA ACT TCC CGC GTT CT	Settembre et al, 2013
Atp6V1d	GCT TCC CTG CAG ACT TCC TT	TAG GCA AGG GTG CGT TCA AT	Settembre et al, 2013
Atp6V1h	GCA GTC TTA TCT TCA GGG GCA	TTT AGC ACA CTG GCT GCC TT	Settembre et al, 2013
Cts a	GTG GTG CTT TGG CTT AAC GG	CTG GCT GGA TCA GAA AGG GG	Settembre et al, 2013
Cts b	TGCGTTCGGTGAGGACATAG	CCAAATGCCCAACAAGAGCC	Settembre et al, 2013
Clcn7	CGC GCG CCA GTC TCA T	TCT CCT TGG GGA AGG TGT GA	Settembre et al, 2013
Cts f	GCT ATG GCA ACC GCT CTA AC	GCT CCA GAT CCA CGG TAC AA	Settembre et al, 2013
Cyclophillin A	CAGACGCCACTGTCGCTTT	TGTCTTTGGAACTTTGTCTGCA A	Joyal et al, 2016
Ffar1 (GPR40)	CCTTCGCTCTCTATGTATCTG CC	GGCTAACAAGTTCAATGGAAA GC	Joyal et al, 2016
Lamp1	TGGCAGGCCTTGCACAT	TCACGATCTGAGAACCATTCG	Settembre et al, 2013
Luciferase	GCATTCCGGTACTGTTGGT	GCAGCGCACTTTGAATCTTG	Addgene
Map1lc3b	GTGGAAGATGTCCGGCTCAT	TGGTCAGGCACCAGGAACTT	Kang et al., 2012
Mcoln1	CCA GTG TCA TGC GTT TCT GC	CGA GCG GAA CTT CAC ATG GT	Settembre et al, 2013
NBR1	CCCCAGATTGGTTTACAAGC	TCCACCGTTTCCTTAACCAC	Pichr-Martel et al., 2018

OPNT1	AGCAAAGAGGTTAAGGAGC GCCTTAAG	CAGCTTCTCCACTTCCTCCTCC AA	Tseng et al., 2016
p62/ Sqstm1	CGC GTG GGT TCG TCC G	CCA AGT CCC CAT CCT CAT CG	Settembre et al, 2013
Pgc1a	ACACCGCAATTCTCCCTTGT	TTTCAGACTCCCGCTTCTCG	Joyal et al, 2016
Ppara	AGAGCCCCATCTGTCCTCT	ACTGGTAGTCTGCAAAACCAA A	Joyal et al, 2016
Sirt1	GAC GAT GAC AGA ACG TCA CA	CGA GGA TCG GTG CCA ATC A	This paper
Sirt3	CCCGGGGCGCT CTA TAC ACA GAA CAT CGA	GGCCGCGGTGCCACTTTACCGA TCAACAT	Joyal et al, 2016
Sirt4	GAC AGA ATA AGA ATG AGC GGA	GGC ACA AAT AAC CCC GAG G	This paper
Sirt5	CTC CGG GCC GAT TCA TTT C	GCG TTC GCA AAA CAC TTC CG	This paper
Tfeb	AACCTAATTGAGAGAAGACG CAGGT	CCTTGAGGATGGTGCCTTTGT	Settembre et al, 2011
Ubqln2	CTCCACACCTACCACCACGA ATA	GCTGCTGCATCTGGTTCTGAAG	Kirkin et al., 2009
Uvrag	CTCAGCAGCGGCGTCTC	AGGAATTCTTAATCACTTCACT CCT	Settembre et al, 2013
Vps 8	CGCAGCCGACAAAGTAGATG	TAAAGCCAGTCCGTGAGACG	Settembre et al, 2013
Vps 11	GACCAAACTGGAGATGCTGT	GCCAACCCATCACTGTCCAA	Settembre et al, 2013
Vps 18	TTCCTGGACCATACTGGCTC T	CCTTCTGTCCATTGCGGTTC	Settembre et al, 2013
Vps 33	GCGCTACATTGCCAGTCTTG	TCTCCATAGACTCCCTCTTCC	Settembre et al, 2013
Vps 35s	ATA AGC TCT GGG TGC GGA TG	ACT GAC TAA GGC GCA CCA AA	Settembre et al, 2013
Wipi	ATT CGG CTC AAC CGA CAG AG	CAG GGC ACA GAG ACC TGT TG	Settembre et al, 2013

Table S4. Description of reagent or resource

REGEANT or RESOURCE	SOURCE	IDENTIFIER
**Antibodies**		
Anti-β Actin	Cell Signaling Technology	Cat#4970, RRID:AB_2223172
Anti-α-Tubulin	Cell Signaling Technology	Cat# 2144, RRID:AB_2210548
Anti-LAMIN B1	Cell Signaling Technology	Cat# 13435, RRID:AB_2737428
Anti-p-AKT (Ser308)	Cell Signaling Technology	Cat# 13038, RRID:AB_2629447
Anti-AKT (pan)	Cell Signaling Technology	Cat#4691, RRID:AB_915783
Anti-phospho TFEB Ser142	Sigma	Cat#: ABE1971-l-25UL
Anti-TFEB	Cell Signaling Technology	Cat# 4240, RRID:AB_11220225
Anti-TFEB	Bethyl Laboratories	Cat# A303-672A RRID:RRID:AB_11204598
Anti-TFEB chip grade	Abcam	Cat# Ab122910 RRID:RRID:AB_10901928
Anti-phospho-GSK-3β (Ser9)	Cell Signaling Technology	Cat# 5558, RRID:AB_10013750
Anti-GSK-3β	Cell Signaling Technology	Cat# 9315, RRID:AB_490890
Anti-phospho p42/44 MAPK (ERK1/2, Thr2020/Tyr204)	Cell Signaling Technology	Cat#4370, RRID:AB_2315112
Anti- p42/44 MAPK (ERK1/2)	Cell Signaling Technology	CST, #4695, RRID:AB_390779
Anti-phospho Calcineurin (Ser197)	Badrilla	Cat#: A010-80
Anti-Pan Calcineurin A	Cell Signaling Technology	Cat#: 2614
Anti-LC3B	Cell Signaling Technology	Cat# 2775, RRID:AB_915950
Anti-ATG5	Cell Signaling Technology	Cat# 12994, RRID:AB_2630393
Anti-p62/ SQSTM1	Cell Signaling Technology	Cat# 5114, RRID:AB_10624872
Anti- p62/ SQSTM1 rodent specific	Cell Signaling Technology	Cat# 23214, RRID:AB_2798858
Anti-FFAR1	Abcam	Cat#: AB211049
Anti-SIRT3	Cell Signaling Technology	Cat# 5490, RRID:AB_10828246
Anti-IDH2	Cell Signaling Technology	Cat#: 12652
Acetylated-Lysine (Ac-K2-100)-HRP	Cell Signaling Technology	Cat#: 6952

Cy5 Anti Peanut Agglutinin (PNA)	Vector Labs	Cat# :CL-1075-1
Lectin (Bandeiraea simplicifolia)	Vector Labs	Cat#: FL-1101 RRID: AB_2336490
Anti-rabbit IgG, HRP-linked Antibody	Cell Signaling Technology	Cat#: 7074S RRID:AB_2099233
Goat Anti-Mouse IgG (H + L)-HRP Conjugate	Biorad	Cat#:1706516, RRID:AB_11125547
Donkey anti-rabbit IgG, Alexa Fluor 488	Invitrogen	Cat#: A-21206, RRID:AB_2535792
DONKEY ANTI-GOAT IGG (H+L), ALEXA FLUOR 488	Invitrogen	Cat# A-11055, RRID:AB_2534102
**Chemicals**		
2-Hydroxypropyl beta-Cyclodextrin (HPβCD)	Sigma-Aldrich	Cat#: H107-5G
D-(+)-Trehalose Dehydrate	Sigma-Aldrich	Cat#: T9531
Ultra Pure Bovine Serum Albumin	Genprice	Cat#: 495-A0100-010
Sodium Palmitate	Sigma-Aldrich	Cat#: P9767-5G
Middle chain triglycerides, MCT	Nestlé Health Science	Cat#: 9521498
GW9508	Cedarlane	Cat#: BML-GP102-0010
Etomoxir Sodium Salt	Cayman chemicals	Cat#: 11969-25
Chloroquine diphosphate salt	Sigma-Aldrich	Cat#: C6628-25G
**RNAscope probes and Plasmids**		
ON-TARGETplus Non-targeting Pool	Dharmacon	Cat#: D-001810-10-05
ON-TARGETplus siRNA – SMARTpool Mouse Ffar1	Dharmacon	Cat#: L-051167-00-0005
FFAR1 RNAscope® Target Probe C2	ACD-Bio techne	Cat#: 300031-C2
RNAscope® Probe - Mm-Tfeb	ACD-Bio techne	Cat#: 434701

TFEB promoter-luciferase reporter	Addgene	Cat#: 66801<br>RRID:Addgene_66801
pcDNA3	Invitrogen	Cat:# V79020
**Critical Commercial Assays**		
QuantiTect Rev.Transcription Kit	Qiagen	Cat#: 205313
Dual-Luciferase® Reporter Assay System	Promega	Cat#: E1910
RNAscope 2.5 Duplex Detection Kit	ACD-Bio techne	Cat#: 322436
Seahorse XFe96 FluxPak	Agilent	Cat#: 102416-100
Other		
RIPA 10X	Cell Signaling	Cat#: 9806S
RNeasy Mini Kit	Qiagen	Cat#: 74106
Protease inhibitor	Cell Signaling	Cat#: 5871S
Phosphatase inhibitor	Cell Signaling	Cat#: 5870S
**Experimental Models:**		
661W	Dr. Al-Ubaidi (University of Oklahoma)	RRID:CVCL_6240
661W-Tfeb-Luciferase	This paper	N/A
C57B1/6	The Jackson Laboratory	IMSR Cat# JAX:000664,<br>RRID:IMSR_JAX:000664
Vldlr $^{-/-}$	The Jackson Laboratory	IMSR Cat# JAX:002529,<br>RRID:IMSR_JAX:002529
CAG-RFP-EGFP-LC3B	The Jackson Laboratory	IMSR Cat# JAX:027139,<br>RRID:IMSR_JAX:027139
CAG-RFP-EGFP-LC3;*Vldlr* $^{-/-}$	This paper	N/A
Vldlr $^{-/-}$; *FFAR1* $^{-/-}$	Lois E.H. Smith (Joyal et al, 2016)	N/A
Sirt3 $^{-/-}$	The Jackson Laboratory	IMSR Cat# JAX:031201,<br>RRID:IMSR_JAX:031201
**Softwares, Data and Code Availability**		
Prism	Graphpad	RRID:SCR_002798
Fiji	Fiji	RRID:SCR_002285
ImageJ	ImageJ	RRID:SCR_003070
Leica Application Suite X	Leica Microsystems	RRID:SCR_013673
Imaris 9.3.1	Bitplane	RRID: SCR_007370

AxioVision 4.6.3.0 software	Zeiss	https://www.micro-shop.zeiss.com
Wave 2.6.0	Agilent Technologies	N/A
Espion V.4.0 software	Diagnosys LLC	N/A
TraceFinder 3.3 and 4.1 software	Thermo Scientific; Waltham, MA	OPTON-30626
Progenesis QI	Nonlinear Dynamics; Newcastle upon Tyne, UK	N/A
RNA-seq data	GEO	GSE110623
dropSeqPipe	https://github.com/Hoohm/dropSeqPipe	Drop-seq_tools-1.13 (http://mccarrolllab.com/dropseq/)
Seurat	Satija R, Farrell JA, Gennert D, Schier AF, Regev A Nat Biotechnol. 2015 May; 33(5):495-502.	http://satijalab.org/seurat/ https://CRAN.R-project.org/package=Seurat

Table S5. Description of statistical analysis

Figures and panel		Group	n	Difference in Variance (F test, P value)	Statistical test	*P* value
Fig 1	A				Descriptive	
	B	WT	3801 cells from 3 retinas		fGSEA	
	C-E	*Vldlr*$^{-/-}$	5642 cells pooled from 3		Descriptive	
	F				Descriptive	
	G *Wipi*	WT	12 retinas pooled in 6 experiments	F = 2.665 *P* = 0.3059 (*Wipi*)	Unpaired two-tailed Student t-test	0.0160
		Vldlr$^{-/-}$	12 retinas pooled in 6 experiments			
	Uvrag	WT	24 retinas pooled in 12 experiments	F = 5.207 *P* = 0.0145 (*Uvrag*)	Unpaired two-tailed Student t-test with Welch's correction	0.007
		Vldlr$^{-/-}$	22 retinas pooled in 11 experiments			
	Atg5	WT	20 retinas pooled in 10 experiments	F = 1.726 *P* = 0.4328 (*Atg5*)	Unpaired two-tailed Student t-test	<0.0001
		Vldlr$^{-/-}$	18 retinas pooled in 9 experiments			
	Atg12	WT	18 retinas pooled in 9 experiments	F = 3.383 *P* = 0.0886 (*Atg12*)	Unpaired two-tailed Student t-test	<0.0001
		Vldlr$^{-/-}$	30 retinas pooled in 15 experiments			
	Map1lc3b	WT	20 retinas pooled in 10 experiments	F = 1.134 *P* = 0.8715 (*Map1lc3b*)	Unpaired two-tailed Student t-test	<0.0001
		Vldlr$^{-/-}$	28 retinas pooled in 14 experiments			

	NBR1	WT	24 retinas pooled in 12 experiments	F = 1.036<br>*P* = 0.9537<br>(*NBR1*)	Unpaired two-tailed Student t-test	<0.0001
		Vldlr$^{-/-}$	24 retinas pooled in 12 experiments			
	OPTN	WT	24 retinas pooled in 12 experiments	F = 2.037<br>*P* = 0.2537<br>(*OPTN*)	Unpaired two-tailed Student t-test	<0.0001
		Vldlr$^{-/-}$	24 retinas pooled in 12 experiments			
	UBQLN2	WT	24 retinas pooled in 12 experiments	F = 2.215<br>*P* = 0.2030<br>(*UBQLN2*)	Unpaired two-tailed Student t-test	0.0008
		Vldlr$^{-/-}$	24 retinas pooled in 12 experiments			
	Atp6V0a1	WT	22 retinas pooled in 11 experiments	F = 1.752<br>*P* = 0.3860<br>(*Atp6V0a1*)	Unpaired two-tailed Student t-test	0.017
		Vldlr$^{-/-}$	24 retinas pooled in 12 experiments			
	Atp6V0b	WT	24 retinas pooled in 12 experiments	F = 8.305<br>*P* = 0.0169<br>(*Atp6V0b*)	Unpaired two-tailed Student t-test with Welch's correction	0.0006
		Vldlr$^{-/-}$	14 retinas pooled in 7 experiments			
	Atp6V0c	WT	22 retinas pooled in 11 experiments	F = 4.139<br>*P* = 0.0958<br>(*Atp6V0c*)	Unpaired two-tailed Student t-test	0.0383
		Vldlr$^{-/-}$	14 retinas pooled in 7 experiments			
	Atp6V0e1	WT	12 retinas pooled in 6 experiments	F = 2.677<br>*P* = 0.2886<br>(*Atp6V0e1*)	Unpaired two-tailed Student t-test	0.0044
		Vldlr$^{-/-}$	20 retinas pooled in 10 experiments			

	Atp6V1a	WT	20 retinas pooled in 10 experiments	F = 119.5<br>*P* <0.0001<br>(*Atp6V1a*)	Unpaired two-tailed Student t-test with Welch's correction	0.0327
		$Vldlr^{-/-}$	20 retinas pooled in 10 experiments			
	Atp6V1b2	WT	16 retinas pooled in 8 experiments	F = 3.313<br>*P* = 0.0749<br>(*Atp6V1b2*)	Unpaired two-tailed Student t-test	0.1770
		$Vldlr^{-/-}$	24 retinas pooled in 12 experiments			
	Atp6V1c1	WT	20 retinas pooled in 10 experiments	F = 24.12<br>*P* <0.0001<br>(*Atp6V1c1*)	Unpaired two-tailed Student t-test with Welch's correction	0.9208
		$Vldlr^{-/-}$	24 retinas pooled in 12 experiments			
	Atp6V1d	WT	20 retinas pooled in 10 experiments	F = 53.92<br>*P* <0.0001<br>(*Atp6V1d*)	Unpaired two-tailed Student t-test with Welch's correction	0.9719
		$Vldlr^{-/-}$	22 retinas pooled in 11 experiments			
	Atp6V1h	WT	22 retinas pooled in 11 experiments	F = 1.042<br>*P* = 0.9560<br>(*Atp6V1h*)	Unpaired two-tailed Student t-test	0.1484
		$Vldlr^{-/-}$	24 retinas pooled in 12 experiments			
	Ctsa	WT	22 retinas pooled in 11 experiments	F = 2.346<br>*P* = 0.1795<br>(*Ctsa*)	Unpaired two-tailed Student t-test	<0.0001
	Ctsb	$Vldlr^{-/-}$	30 retinas pooled in 15 experiments			
		WT	24 retinas pooled in 12 experiments	F = 1.317<br>*P* = 0.6549<br>(*Ctsb*)	Unpaired two-tailed Student t-test	<0.0001

	Ctsf	*Vldlr*$^{-/-}$	30 retinas pooled in 15 experiments			
		WT	16 retinas pooled in 8 experiments	F = 1.142 *P* = 0.8099 (*Ctsf*)	Unpaired two-tailed Student t-test	0.0171
	Clcn7	*Vldlr*$^{-/-}$	24 retinas pooled in 12 experiments			
		WT	18 retinas pooled in 9 experiments	F = 1.944 *P* = 0.2758 (*Clcn7*)	Unpaired two-tailed Student t-test	<0.0001
	Lamp1	*Vldlr*$^{-/-}$	28 retinas pooled in 14 experiments			
		WT	18 retinas pooled in 9 experiments	F = 1.276 *P* = 0.6591 (*Lamp1*)	Unpaired two-tailed Student t-test	0.0003
	Mcoln1	*Vldlr*$^{-/-}$	30 retinas pooled in 15 experiments			
		WT	22 retinas pooled in 11 experiments	F = 2.607 *P* = 0.1084 (*Mcoln1*)	Unpaired two-tailed Student t-test	0.0001
	Vps8	*Vldlr*$^{-/-}$	28 retinas pooled in 14 experiments			
		WT	18 retinas pooled in 9 experiments	F = 3.350 *P* = 0.0774 (*Vps8*)	Unpaired two-tailed Student t-test	0.0002
	Vps11	*Vldlr*$^{-/-}$	22 retinas pooled in 11 experiments			
		WT	16 retinas pooled in 8 experiments	F = 59.05 *P* <0.0001 (*Vps11*)	Unpaired two-tailed Student t-test with Welch's correction	<0.0001
	Vps18	*Vldlr*$^{-/-}$	24 retinas pooled in 12 experiments			
		WT	14 retinas pooled in 7 experiments	F = 1.881 *P* = 0.3603 (*Vps18*)	Unpaired two-tailed Student t-test	0.0002

	Vps33	*Vldlr*$^{-/-}$	22 retinas pooled in 11 experiments			
		WT	16 retinas pooled in 8 experiments	F = 2.725<br>*P* = 0.1469<br>(*Vps33*)	Unpaired two-tailed Student t-test	<0.0001
	Vps35	*Vldlr*$^{-/-}$	22 retinas pooled in 11 experiments			
		WT	16 retinas pooled in 8 experiments	F = 2.599<br>*P* = 0.1521<br>(*Vps35*)	Unpaired two-tailed Student t-test	<0.0001
		Vldlr$^{-/-}$	24 retinas pooled in 12 experiments			
	H				Descriptive	
	I	WT	8 retinas	One-way ANOVA,<br>F = 6.597<br>*p* = 0.0541<br>(RFP)<br><br>F = 7.768<br>*p* = 0.0383<br>(Yellow)	Unpaired two-tailed Student t-test (RFP)<br><br>Unpaired two-tailed Student t-test with Welch's correction (Yellow)	<0.0001 (RFP)<br>0.0003 (Yellow)
		Vldlr$^{-/-}$	6 retinas			
	J-K	WT	8 retinas	F = 3.273<br>*p* = 0.1462	Unpaired two-tailed Student t-test	0.0007
		Vldlr$^{-/-}$	7 retinas			
	L	WT	12 retinas pooled in 6 experiments	F = 39.05<br>*p* = 0.0010	Unpaired two-tailed Student t-test with Welch's correction	0.0438
		Vldlr$^{-/-}$	12 retinas pooled in 6 experiments			
Fig 3	A	WT	5 retinas	One-way ANOVA,<br>F = 45.36<br>*p* = 0.6786<br>(RFP)<br>F = 11.19	Tukey's Multiple Comparison Test	<0.0001<br><br>0.0009

				p = 0.7666 (yellow)		
		Vldlr$^{+/–}$	8 retinas			
		Vldlr$^{–/–}$	6 retinas			
	B	WT	10 retinas pooled in 5 experiments	One-way ANOVA, F = 82.94 *p* <0.0001	Tukey's Multiple Comparison Test	WT vs *Vldlr*$^{+/–}$: <0.001<br>WT vs *Vldlr*$^{-/–}$: <0.0001<br>*Vldlr*$^{+/–}$ vs *Vldlr*$^{-/–}$: <0.0001
		Vldlr$^{+/–}$	8 retinas pooled in 4 experiments			
		Vldlr$^{–/–}$	8 retinas pooled in 4 experiments			
	C	WT	7 mice	One-way ANOVA, F = 47.32 *p* <0.0001	Dunnett's Multiple Comparison Test	WT vs *Vldlr*$^{+/–}$: ns<br>WT vs *Vldlr*$^{-/–}$: <0.0001
		Vldlr$^{+/–}$	8 mice			
		Vldlr$^{–/–}$	13 mice			
	D	WT fed	14 retinas	F = 34.81<br><br>*p* <0.0001 (RFP)<br><br>F = 30.91 *p* <0.0001 (yellow)	Tukey's Multiple Comparison Test	(RFP)<br>WT fed vs WT + MCT: <0.01<br><br>WT fed vs WT starved : <0.0001<br><br>WT starved vs WT starved + MCT: <0.0001<br><br>(Yellow)<br>WT fed vs WT + MCT: <0.01<br><br>WT fed vs WT starved : <0.0001

						WT starved vs WT starved + MCT: <0.001
		WT fed	6 retinas			
		WT starved	8 retinas			
		WT + MCT	10 retinas			
	E<br>TG	WT<br>Veh	15 mice	F = 1.402<br>*p* = 0.5331	Unpaired two-tailed Student t-test	WT veh vs WT + MCT : <0,0001
		WT<br>MCT	16 mice			
		Vldlr$^{-/-}$<br>Veh	9 mice	F = 7.512<br>*p* = 0.0080	Unpaired two-tailed Student t-test with Welch's correction	WT veh vs WT + MCT : 0.0307
		Vldlr$^{-/-}$<br>MCT	13 mice			
	F	WT	12 retinas pooled in 6 experiments	One-way ANOVA,<br>F = 5.234<br>*p* = 0.2290	Dunnett's Multiple Comparison Test	WT vs *Vldlr*$^{+/-}$: ns<br>WT vs *Vldlr*$^{-/-}$: 0.0189
		Vldlr$^{+/-}$	12 retinas pooled in 6 experiments			
		Vldlr$^{-/-}$	12 retinas pooled in 6 experiments			
	G<br>TFEB WB	WT<br>Veh	8 retinas pooled in 4 experiments	F = 3.275<br>*p* = 0.1777	Unpaired two-tailed Student t-test	0.0026
		WT<br>MCT	16 retinas pooled in 8 experiments			
		Vldlr$^{-/-}$<br>Veh	8 retinas pooled in 4 experiments	F = 2.402<br>*p* = 0.5053	Unpaired two-tailed Student t-test	0.0358
		Vldlr$^{-/-}$<br>MCT	16 retinas pooled in 8 experiments			
Fig 4	A				Descriptive	

	B-C	WT	9000 cells pooled from 3 retinas per group		GSEA	as described on figure
		Vldlr$^{-/-}$				
	D<br>CaN (left panel)	WT	24 retinas pooled in 12 experiments	One-way ANOVA,<br>F = 69.96<br>p <0.0001	Tukey's Multiple Comparison Test	WT vs *Vldlr*$^{-/-}$: <0.05<br>WT vs *Vldlr*$^{-/-}$/ *Ffar1*$^{-/-}$: <0.001<br>*Vldlr*$^{-/-}$ vs *Vldlr*$^{-/-}$/ *Ffar1*$^{-/-}$: <0.001
		Vldlr$^{-/-}$	22 retinas pooled in 11 experiments			
		Vldlr$^{-/-}$/ *Ffar1*$^{-/-}$	24 retinas pooled in 12 experiments			
	D<br>p-CaN (right panel)	WT	24 retinas pooled in 12 experiments	One-way ANOVA,<br>F = 15.49<br>p <0.0001	Tukey's Multiple Comparison Test	WT vs *Vldlr*$^{-/-}$: <0.05<br>WT vs *Vldlr*$^{-/-}$/ *Ffar1*$^{-/-}$: <0.05<br>*Vldlr*$^{-/-}$ vs *Vldlr*$^{-/-}$/ *Ffar1*$^{-/-}$: <0.001
		Vldlr$^{-/-}$	24 retinas pooled in 12 experiments			
		Vldlr$^{-/-}$/ *Ffar1*$^{-/-}$	24 retinas pooled in 12 experiments			
	E<br>Total Tfeb (left panel)	WT	36 retinas pooled in 18 experiments	One-way ANOVA,<br>F = 11.67<br>p <0.0001	Tukey's Multiple Comparison Test	WT vs *Vldlr*$^{-/-}$: <0.05<br>WT vs *Vldlr*$^{-/-}$/ *Ffar1*$^{-/-}$: <0.05<br>*Vldlr*$^{-/-}$ vs *Vldlr*$^{-/-}$/ *Ffar1*$^{-/-}$: <0.001
		Vldlr$^{-/-}$	22 retinas pooled in 11 experiments			

		Vldlr$^{-/-}$/ *Ffar1*$^{-/-}$	24 retinas pooled in 12 experiments			
	E (p- Tfeb, right panel)	WT	22 retinas pooled in 11 experiments	One-way ANOVA, F = 33.04 *p* <0.0001	Tukey's Multiple Comparison Test	WT vs *Vldlr*$^{-/-}$: <0.001 WT vs *Vldlr*$^{-/-}$/ *Ffar1*$^{-/-}$: <0.05 *Vldlr*$^{-/-}$ vs *Vldlr*$^{-/-}$/ *Ffar1*$^{-/-}$: <0.001
		Vldlr$^{-/-}$	18 retinas pooled in 9 experiments			
		Vldlr$^{-/-}$/ *Ffar1*$^{-/-}$	22 retinas pooled in 11 experiments			
	F	WT	12 retinas pooled in 6 experiments	One-way ANOVA, F = 14.44 *p* = 0.0002	Bonferroni's Multiple Comparison Test	WT vs *Vldlr*$^{-/-}$: <0.01 WT vs *Vldlr*$^{-/-}$/ *Ffar1*$^{-/-}$: ns
		Vldlr$^{-/-}$	14 retinas pooled in 7 experiments			
		Vldlr$^{-/-}$/ *Ffar1*$^{-/-}$	16 retinas pooled in 8 experiments			
	G	WT	16 retinas pooled in 8 experiments	One-way ANOVA, F = 8.929 *p* <0.0001	Bonferroni's Multiple Comparison Test	WT vs *Vldlr*$^{-/-}$: <0.01 WT vs *Vldlr*$^{-/-}$/ *Ffar1*$^{-/-}$: ns
		Vldlr$^{-/-}$	20 retinas pooled in 10 experiments			
		Vldlr$^{-/-}$/ *Ffar1*$^{-/-}$	8 retinas pooled in 4 experiments			
	H	WT	18 retinas pooled in 9 experiments	Non Gaussian distribution	Kruskal-Wallis with Dunn's Multiple Comparison Test	WT vs *Vldlr*$^{-/-}$: <0.001 WT vs *Vldlr*$^{-/-}$/ *Ffar1*$^{-/-}$: ns

		Vldlr$^{-/-}$	20 retinas pooled in 10 experiments			
		Vldlr$^{-/-}$/ *Ffar1*$^{-/-}$	8 retinas pooled in 4 experiments			
Fig 5	A	Human control (macula r hole)	4 vitreous	As described on the figure	MSEA from Metabo Analyst	As described on the figure
		Human vitreous sample from AMD patients	6 vitreous			
		Human vitreous sample from RAP patients	8 vitreous			
		WT	7 retinas			
		Vldlr$^{-/-}$	8 retinas			
	B	Human control (macula r hole)	4 vitreous		Descriptive	
		Human vitreous sample from AMD patients	6 vitreous			
		Human vitreous sample from RAP patients	8 vitreous			
		WT	7 retinas			
		Vldlr$^{-/-}$	8 retinas			
	C				Descriptive	
	D	WT	12 retinas pooled in 6 experiments	F = 2.197, p = 0.4080 (*Sirt1*)	Unpaired two-tailed Student t-test	0.0266 (*Sirt1*) 0.0321

				F = 50.58, p = 0.0006 (*Sirt3*) F = 3.426, p = 0.2027 (*Sirt4*) F = 2.976, p = 0.2566 (*Sirt5*)	and Unpaired two-tailed Student t-test with Welch's correction (*Sirt3*)	(*Sirt3*) 0.6219 (*Sirt4*) 0.0662 (*Sirt5*)
		Vldlr$^{-/-}$	12 retinas pooled in 6 experiments			
	E	WT	6 retinas pooled in 3 experiments	F = 1.115, p = 0.9456	Unpaired two-tailed Student t-test	0.0019
		Vldlr$^{-/-}$	6 retinas pooled in 3 experiments			
	F	WT	9 retinas pooled in 3 experiments	One-way ANOVA, F = 17.39, p = 0.0032	Dunnett's Multiple Comparison Test	WT vs *Vldlr*$^{-/-}$ lesions <0.01 WT vs *Vldlr*$^{-/-}$ no lesions: <0.01
		Vldlr$^{-/-}$	9 retinas pooled in 3 experiments			
	G	WT	3801 cells from 3 retinas		GSEA	As decribed on figure
		Vldlr$^{-/-}$	5642 cells from 3 retinas			
Fig 6	A-B	Control (BSA)	6 wells	One-way ANOVA, F = 74.53, p <0.0001	Tukey's Multiple Comparison Test	Ctl (BSA) veh vs palm-veh : <0.0001 Ctl (BSA) Eto vs palm + Eto : <0.001 Ctl (BSA) veh vs Ctl (BSA)Eto: <0.0001 Palm-veh vs palm-Eto: <0.0001
		Palmitate	5 wells			

		Control + Etomoxir	6 wells			
		Palmitate+ Etomoxir	6 wells			
	C-D	siScrm + BSA	5 wells	One-way ANOVA, F = 16.36 *p* <0.0001	Tukey's Multiple Comparison Test	siScrm + BSA vs siScrm + Palm : <0.01 si*Ffar1* + BSA vs si*Ffar1* + Palm : ns siScrm + BSA vs si*Ffar1* + BSA : ns siScrm + Palm vs si*Ffar1* + Palm : <0.01
		siScrm + Palmitate	5 wells			
		si*Ffar1* + BSA	6 wells			
		si*Ffar1* + Palmitate	6 wells			
	E	$Vldlr^{-/-}$	34 retinas	One-way ANOVA, F = 29.57 *p* <0.0001	Tukey's Multiple Comparison Test	$Vldlr^{-/-}$ vs $Vldlr^{-/-}$ + MCT :<0.001 $Vldlr^{-/-}$ vs $Vldlr^{-/-}$/ $Ffar1^{-/-}$: <0.001 $Vldlr^{-/-}$ + MCT vs $Vldlr^{-/-}$/ $Ffar1^{-/-}$ + MCT: <0.001 $Vldlr^{-/-}$/ $Ffar1^{-/-}$vs $Vldlr^{-/-}$/ $Ffar1^{-/-}$+ MCT: ns
		$Vldlr^{-/-}$ + MCT	8retinas			

		Vldlr$^{-/-}$/ *Ffar1*$^{-/-}$	10 retinas			
		Vldlr$^{-/-}$/ *Ffar1*$^{-/-}$ + MCT	5 retinas			
Fig 7	A	Vehicle	6 retinas	F = 10.13<br>p = 0.0224 (RFP)<br>F = 5.500<br>p = 0.0813 (Yellow)	Unpaired two-tailed Student t-test with Welch's correction (RFP)<br>Unpaired two-tailed Student t-test (Yellow)	0.0055 (RFP)<br>0.0016 (Yellow)
		HPβCD	7 retinas			
	B<br>*Tfeb*	Vehicle	18 retinas pooled in 9 experiments	F = 20.91<br>p = 0.0004	Unpaired two-tailed Student t-test with Welch's correction	0.0375
		HPβCD	14 retinas pooled in 7 experiments			
	Atg5	Vehicle	22 retinas pooled in 11 experiments	F = 1.206<br>p = 0.8065	Unpaired two-tailed Student t-test	0.0369
		HPβCD	22 retinas pooled in 11 experiments			
	Pgc1α	Vehicle	22 retinas pooled in 11 experiments	F = 5.388<br>p = 0.0254	Unpaired two-tailed Student t-test with Welch's correction	0.0004
		HPβCD	18 retinas pooled in 9 experiments			
	Pparα	Vehicle	24 retinas pooled in 12 experiments	F = 3.113<br>p = 0.0906	Unpaired two-tailed Student t-test	0.0269
		HPβCD	18 retinas pooled in 9 experiments			

	Sirt3	Vehicle	20 retinas pooled in 10 experiments	F = 2.370<br>*p* = 0.2147	Unpaired two-tailed Student t-test	0.0069
		HPβCD	20 retinas pooled in 10 experiments			
	C	BSA (Ctl)	7 wells	One-way ANOVA,<br>F = 21.08<br>*p* <0.0001	Tukey's Multiple Comparison Test	Ctl (BSA) veh vs palm + veh : <0.001<br>BSA + HPβCD vs Palm + HPβCD : <0.01<br>Ctl (BSA) veh vs BSA + HPβCD : <0.05<br>Palm + veh vs Palm + HPβCD : <0.01<br>Ctl (BSA) veh vs Palm + HPβCD : ns
		Palmitate	8 wells			
		BSA + HPβCD	6 wells			
		Palmitate + HPβCD	6 wells			
	D	BSA (Ctl)	7 wells	One-way ANOVA,<br>F = 31.32<br>*p* <0.0001	Tukey's Multiple Comparison Test	Ctl (BSA) veh vs palm + veh : <0.0001<br>BSA + CQ vs Palm + CQ : <0.01<br>Ctl (BSA) veh vs BSA + CQ : <0.0001<br>Palm + veh vs Palm + CQ : <0.001<br>Ctl (BSA) veh vs Palm + CQ : <0.0001
		Palmitate	8 wells			

		BSA + Chloroquine (CQ)	6 wells			
		Palmitate + Chloroquine (CQ)	6 wells			
	E	Vehicle	7 retinas	F = 1.691<br>*p* = 0.5808	Unpaired two-tailed Student t-test	0.0031
		HPβCD	6 retinas			

Supplemental Figures and panel		Group	n	Difference in Variance (F test, P value)	Statistical test	*P* value
SF1	A-E	WT	8 retinas		Descriptive	
	D-E	WT	3 retinas		Descriptive	
		Vldlr$^{-/-}$	3 retinas			
SF2	A	*WT*	3801 cells from 3 retinas			
	B	*Human*				From GEO: GSE148077
	C	WT	3801 cells from 3 retinas		GSEA	As decribed on figure
		Vldlr$^{-/-}$	5642 cells from 3 retinas			
SF3	A				Descriptive	
	B	WT	12 retinas pooled in 6 experiments	F = 7.497<br>*p* = 0.0061	Tukey's Multiple Comparison Test	WT vs *Vldlr*$^{-/-}$: <0.05<br>WT vs *Vldlr*$^{-/-}$/ *Ffar1*$^{-/-}$: <0.01<br>*Vldlr*$^{-/-}$ vs *Vldlr*$^{-/-}$/ *Ffar1*$^{-/-}$: ns
		Vldlr$^{-/-}$	10 retinas pooled in 5 experiments			
		Vldlr$^{-/-}$/ *Ffar1*$^{-/-}$	12 retinas pooled in 6 experiments			
	C	WT	6 retinas pooled in 3 experiments	F = 7.570<br>*p* = 0.0229	Tukey's Multiple Comparison Test	WT vs *Vldlr*$^{-/-}$: <0.05<br>WT vs *Vldlr*$^{-/-}$/ *Ffar1*$^{-/-}$: <0.05<br>*Vldlr*$^{-/-}$ vs *Vldlr*$^{-/-}$/ *Ffar1*$^{-/-}$: ns

		Vldlr$^{-/-}$	6 retinas pooled in 3 experiments			
		Vldlr$^{-/-}$/ *Ffar1*$^{-/-}$	6 retinas pooled in 3 experiments			
	D	WT	12 retinas pooled in 6 experiments	F = 11.36 *p* = 0.0010	Tukey's Multiple Comparison Test	WT vs *Vldlr*$^{-/-}$: <0.01 WT vs *Vldlr*$^{-/-}$/ *Ffar1*$^{-/-}$: <0.01 *Vldlr*$^{-/-}$ vs *Vldlr*$^{-/-}$/ *Ffar1*$^{-/-}$: ns
		Vldlr$^{-/-}$	12 retinas pooled in 6 experiments			
		Vldlr$^{-/-}$/ *Ffar1*$^{-/-}$	12 retinas pooled in 6 experiments			
	E	WT	12 retinas pooled in 6 experiments	F = 10.97 *p* = 0.0012	Tukey's Multiple Comparison Test	WT vs *Vldlr*$^{-/-}$: <0.05 WT vs *Vldlr*$^{-/-}$/ *Ffar1*$^{-/-}$: <0.01 *Vldlr*$^{-/-}$ vs *Vldlr*$^{-/-}$/ *Ffar1*$^{-/-}$: ns
		Vldlr$^{-/-}$	12 retinas pooled in 6 experiments			
		Vldlr$^{-/-}$/ *Ffar1*$^{-/-}$	12 retinas pooled in 6 experiments			
	F	WT	6 retinas pooled in 3 experiments	F = 18.25 *p* = 0.0028	Tukey's Multiple Comparison Test	WT vs *Vldlr*$^{-/-}$: <0.01 WT vs *Vldlr*$^{-/-}$/ *Ffar1*$^{-/-}$: <0.01

						Vldlr$^{-/-}$ vs *Vldlr*$^{-/-}$/ *Ffar1*$^{-/-}$: ns
		Vldlr$^{-/-}$	6 retinas pooled in 3 experiments			
		Vldlr$^{-/-}$/ *Ffar1*$^{-/-}$	6 retinas pooled in 3 experiments			
	G	WT	20 retinas pooled in 10 experiments	F = 22.16 *p* <0.0001	Tukey's Multiple Comparison Test	WT vs *Vldlr*$^{-/-}$: <0.05 WT vs *Vldlr*$^{-/-}$/ *Ffar1*$^{-/-}$: <0.01 *Vldlr*$^{-/-}$ vs *Vldlr*$^{-/-}$/ *Ffar1*$^{-/-}$: <0.001
		Vldlr$^{-/-}$	18 retinas pooled in 9 experiments			
		Vldlr$^{-/-}$/ *Ffar1*$^{-/-}$	20 retinas pooled in 10 experiments			
	H	WT	18 retinas pooled in 9 experiments	Non Gaussian distribution	Kruskal-Wallis with Dunn's Multiple Comparison Test	WT vs *Vldlr*$^{-/-}$: <0.05 WT vs *Vldlr*$^{-/-}$/ *Ffar1*$^{-/-}$: ns *Vldlr*$^{-/-}$ vs *Vldlr*$^{-/-}$/ *Ffar1*$^{-/-}$: <0.001
		Vldlr$^{-/-}$	18 retinas pooled in 9 experiments			
		Vldlr$^{-/-}$/ *Ffar1*$^{-/-}$	8 retinas pooled in 4 experiments			

SF4	A	siScrm + Vehicle	9 independent experiments	F = 4.998<br>*p* = 0.0056	Bonferroni's Multiple Comparison Test	siScrm + Veh vs siScrm + Palm : <0.05<br>si*Ffar1* + Veh vs si*Ffar1* + Palm : ns
		siScrm + Palmitate	9 independent experiments			
		si*Ffar1* + Vehicle	10 independent experiments			
		si*Ffar1* + Palmitate	10 independent experiments			
	B	siScrm + Vehicle	7 independent experiments	F = 11.50<br>*p* <0.0001	Bonferroni's Multiple Comparison Test	siScrm + Veh vs siScrm + Palm : <0.05<br>si*Ffar1* + Veh vs si*Ffar1* + Palm : ns
		siScrm + Palmitate	7independent experiments			
		si*Ffar1* + Vehicle	7 independent experiments			
		si*Ffar1* + Palmitate	7 independent experiments			
	C	siScrm + Vehicle	11 experiments	F = 13.90<br>*p* <0.0001	Bonferroni's Multiple Comparison Test	siScrm + Veh vs siScrm + MCT : <0.01<br>si*Ffar1* + Veh vs si*Ffar1* + MCT : ns
		siScrm + MCT	13 experiments			

		si*Ffar1* + Vehicle	18 experiments			
		si*Ffar1* + MCT	18 experiments			
	D				Descriptive	
	E	Fed	4 independent experiments, 48 cells	Non parametric Test	Kruskal-Wallis with Dunn's Multiple Comparison Test	Starved vs starved GW9508 <0.001 Starved vs starved MCT <0.001 Starved MCT vs starved GW9508 <0.001
		Starved	4 independent experiments, 52 cells			
		GW9508	4 independent experiments, 43 cells			
		MCT	4 independent experiments, 76 cells			
	F	Vehicle	12 retinas pooled in 6 experiments	$F = 6.797$ $p = 0.0592$ (cytoplasm) $F = 3.011$ $p = 0.2579$ (nucleus)	Unpaired two-tailed Student t-test	0.0071 (cytoplasm) 0.0007 (nucleus)
		GW9508	10 retinas pooled in 5 experiments			
SF5	A-D	WT	15 retinas		PLSDA	
		Human control	5 vitreous			

		(macular hole)				
		Vldlr$^{-/-}$	18 retinas			
		Human vitreous sample from AMD patients	7 vitreous			
		Human vitreous sample from RAP patients	8 vitreous			
SF6	A TFEB	siScrm	12 experiments	F = 2.136 *p* = 0.5793	Unpaired two-tailed Student t-test	0.0063
		si*Tfeb*	4 experiments			
	ATG5	siScrm	4 experiments	F = 4.382 *p* = 0.2562	Unpaired two-tailed Student t-test	0.0255
		si*Tfeb*	4 experiments			
	LC3B-II	siScrm	8 experiments	F = 1.010 *p* = 0.9897	Unpaired two-tailed Student t-test	0.0052
		si*Tfeb*	8 experiments			
	SIRT3	siScrm	19 experiments	F = 1.173 *p* = 0.7454	Unpaired two-tailed Student t-test	0.0007
		si*Tfeb*	19 experiments			
	B *Pparα*	siScrm	3 Independent experiments	F = 2.487 *p* = 0.3400	Unpaired two-tailed Student t-test	0.0280
		si*Tfeb*	3 Independent experiments			
	C *Pgc1α*	siScrm	3 Independent experiments	F = 4.424 *p* = 0.1284	Unpaired two-tailed Student t-test	0.0007
		si*Tfeb*	3 Independent experiments			
	D	Control	3 Independant tests	F = 1.318 *p* = 0.8630 (*Sirt1*)	Unpaired two-tailed Student t-test	0.2162 (*Sirt1*) 0.0175

				F = 8.293<br>p = 0.2048<br>(*Sirt3*)<br>F = 254.1<br>p = 0.0886<br>(*Sirt4*)<br>F = 43.27<br>p = 0.2137<br>(*Sirt5*)		(*Sirt3*)<br>0.5014<br>(*Sirt4*)<br>0.4859<br>(*Sirt5*)
		GW9508	3 Independant tests			
	E	Vehicle	3 Independant tests	F = 1.887<br>p = 0.9154	Unpaired two-tailed Student t-test	0.0142
		MCT	3 Independant tests			
	F	Vehicle	6 wells	F = 1.569<br>p = 0.6462<br>(ATP production)	Unpaired two-tailed Student t-test	<0.0001
		GW9508	11 wells			
	G	Veh	6 retinas pooled in 3 experiments	F = 17.07<br>p = 0.1107	Unpaired two-tailed Student t-test	0.0003
		GW9508	6 retinas pooled in 3 experiments			
	H	WT	14 retinas pooled in 7 experiments	F = 8.069<br>p = 0.1107	Dunnett's Multiple Comparison Test	WT vs *Vldlr*$^{-/-}$: <0.01<br>WT vs *Vldlr*$^{-/-}$/ *Ffar1*$^{-/-}$: <0.05
		Vldlr$^{-/-}$	20 retinas pooled in 10 experiments			
		Vldlr$^{-/-}$/ *Ffar1*$^{-/-}$	12 retinas pooled in 6 experiments			
	I	WT	16 retinas pooled in 8 experiments	F = 4.651<br>p = 0.0115	Bonferroni's Multiple Comparison Test	WT vs *Vldlr*$^{-/-}$: <0.01

						WT vs *Vldlr*$^{-/-}$/ *Ffar1*$^{-/-}$: ns
		Vldlr$^{-/-}$	20 retinas pooled in 10 experiments			
		Vldlr$^{-/-}$/ *Ffar1*$^{-/-}$	8 retinas pooled in 4 experiments			
	J				Descriptive	
SF7	A	Vehicle	6 retinas	F = 2.119 *p* = 0.4294 (RFP) F = 1.082 *p* = 0.59333 (Yellow)	Unpaired two-tailed Student t-test	<0.0001 (RFP) <0.0001 (Yellow)
		Trehalose	6 retinas			
	B *Tfeb*	Vehicle	10 retinas pooled in 5 experiments	F = 48.41 *p* = 0.0024	Unpaired two-tailed Student t-test with Welch's correction	0.0163
		Trehalose	10 retinas pooled in 5 experiments			
	Atg5	Vehicle	12 retinas pooled in 6 experiments	F = 2.331 *p* = 0.4326	Unpaired two-tailed Student t-test	0.0115
		Trehalose	10 retinas pooled in 5 experiments			
	Pgc1α	Vehicle	10 retinas pooled in 5 experiments	F = 1.535 *p* = 0.6710	Unpaired two-tailed Student t-test	0.0434
		Trehalose	10 retinas pooled in 5 experiments			
	Pparα	Vehicle	10 retinas pooled in 5 experiments	F = 34.46 *p* = 0.0047	Unpaired two-tailed Student t-test with Welch's correction	0.0074
		Trehalose	10 retinas pooled in 5 experiments			

	Sirt3	Vehicle	10 retinas pooled in 5 experiments	F = 5.456 *p* = 0.1291	Unpaired two-tailed Student t-test	< 0.0001
		Trehalose	10 retinas pooled in 5 experiments			
	C	Vehicle	8 mice	F = 10.74 *p* = 0.0101	Unpaired two-tailed Student t-test with Welch's correction	0.00133
		HPβCD	7 mice			
	D	Vehicle	5 independent experiments (x triplicates, 11 readings)	F = 1.382 *p* = 0.7763	Unpaired two-tailed Student t-test	0.0079
		HPβCD	6 independent experiments (x triplicates, 11 readings)			
	E	Vehicle	6 Independent experiments	F = 1.156 *p* = 0.8771	Unpaired two-tailed Student t-test	0.0019
		HPβCD	6 Independent experiments			
	F-G	WT	14 retinas	F = 1.386 *p* = 0.5516	Unpaired two-tailed Student t-test	0.0012
		Trehalose	15 retinas			

SUPPLEMENTARY METHODS

Human retina collection

All patients previously diagnosed with type 3 neovascular membrane (or retinal angiomatous proliferation - RAP) were treated with Bevacizumab 1.25 mm/0.05ml and followed by a single vitreoretinal surgeon (F.A. Rezende; HMR). Control patients undergoing surgical treatment for nonvascular pathology (Macular Hole) by the same surgeon (F.A.R.). In an operating room setting, patients underwent surgery under local retro/peribulbar anesthesia. A 5% povidone-iodine solution was used to clean the periocular skin. Vitrectomy was performed through 25-gauge valved cannulas (Alcon) and undiluted samples were collected at the beginning of the procedure. For patients with RAP and wet AMD patients had their undiluted vitreous tap samples collected with a 25-gauge sutureless Retrector® system (Insight Instruments, Stuart, FL) just prior to bevacizumab 1.25 mg/0.05 ml intravitreal injection. Vitreous humour was aliquoted in steril tubes and immediately frozen on dry ice. Criteria for differentiating neovascular membrane (NVM) types 1 and 2 (wet AMD) vs type 3 (RAP) was based on spectral-domain optical coherence tomography (OCT) radial scans and OCT-angiography 3x3 mm scans. NVM Type 1 is under the retinal pigment epithelium (RPE), NVM Type 2 is above the RPE on the subretinal space (both named wet AMD) and NVM Type 3 (RAP) was intraretinal neovascularization only, prior to extending towards the subretinal space. All healthy control subjects had no history of hypertension, cigarette smoking or diabetes and presented full-thickness macular hole without clinical signs of vascular retinopathies.

Mice retina collection

All retinas and eyes were collected at the same time of day (10 am) in order to limit variation in autophagy caused by circadian rhythm, with the exception of experiments that required starvation (8 hours) and their corresponding controls that were performed during the day. Littermate controls were used for each experiment.

Mice drug treatments

Vldlr$^{-/-}$, *Ffar1*$^{-/-}$/*Vldlr*$^{-/-}$ and C57 control mice were gavaged with MCT or NaCl 0.9% (40 μl/once a day, P8 to P16); other mice were injected intraperitoneally (i.p., once a day, P8 to P16) with HPβCD (8g/kg), trehalose (2g/kg) or NaCl 0.9% (vehicle). Following treatment, mice were dark-adapted and fasted for 8-hours before tissue collection. *Sirt3*$^{-/-}$ mice were raised in dark and injected with GW9508 or NaCl 0.9% (i.p twice a day) or with chloroquine (75mg/Kg, one a day) from P8 to P16 before eye enucleation.

Cell drug treatments

Cells were equally distributed into 6-, 12- or 24-well plates and cultured to 80% confluence. Cells were incubated for 17h with BSA-Palmitate (0.2mM), MCT (0.4%, Nestlé), HPβCD (1mM, Sigma-Aldrich), chloroquine (40μM, Sigma-Aldrich), GW9508 (30μM, Cedarlane) or their respective vehicle. Non aqueous solution were dissolved in DMSO prior to experiment. Final concentration of DMSO in culture cell media was maintained under 0.01%. Photoreceptor 661W cells were collected for protein or mRNA extraction using RIPA buffer (Cell Signaling), NE-PER™ Nuclear and Cytoplasmic Extraction Reagents (ThermoFisher Scientific) or RNeasy kit (Qiagen), according to the manufacturer's protocol.

siRNA cell transfection

661W cells were transfected with siRNA against *Ffar1* (40nM, Dharmacon), against *Tfeb* (65nM, Dharmacon), or scrambled sequences using lipofectamine 2000 (Thermo-Fischer Scientific); maximal gene depletion was observed 48 hours following transfection. Cells were then collected for mRNA and protein or treated with BSA (control) or BSA-palmitate (0.2mM) for 17h before protein extraction with RIPA1X buffer (Cell Signaling). For seahorse experiments, cells were trypsinized after FA treatment and cultured in 96 well plate (20000 cells/well) to perform OCR experiments.

Tfeb-luciferase reporter construction

Tfeb luciferase reporter cell lines and controls were generated by transfecting a *Tfeb* promoter-luciferase reporter plasmid (2,5 μg/ μl, plasmid #66801, Addgene) and pcDNA3 plasmid (1 μ g/ μl, Invitrogen) using Lipofectamine 2000 (Thermo-Fischer Scientific) according to the manufacturer's protocol. Clones were selected using G418-genetycin (Sigma-Aldrich) antibiotic (3mg/ml). Individual clones expressing the Tfeb promoter-luciferase plasmid were screened by PCR using primers detecting the luciferase (Foward: GCATTCCGGTACTGTTGGT; Reverse: GCAGCGCACTTTGAATCTTG). Selected cells were stored in liquid nitrogen until use.

Dual luciferase Assay

Cells with stable expression of Tfeb-Luciferase reporter were, if necessary, transfected with siRNA against *Ffar1* as described above and seeded ($3x10^4$) in 24-well plates and transfected with Renilla (pRL-CMV Vector, Promega) for 24 hours prior to stimulation with MCT (0.4%, Nestlé), HPβCD (1mM, Sigma-Aldrich) or their respective vehicle. Cells were starved 4 hours with serum-free DMEM without L-Glutamine (Stock 319-025-CL, Wisent), in addition to treatments. Luciferase activity was measured using the Dual-Luciferase® Reporter Assay System (Promega) according to the manufacturer's protocol. Each independent experiment was repeated at least three times and normalized using the Renilla signal.

Immunoprecipitation

Cells were cultured to 80% confluence and treated 17h with MCT (0.4%, Nestlé), washed with PBS and their protein were collected in RIPA buffer containing protease inhibitors (ratio 1:100, Sigma-Aldrich). For immunoprecipitations, protein lysates (1 μg/ml; 200 μl) were incubated overnight with IDH2 primary antibody (1/50, Cell Signaling). Protein A-agarose beads (20 µl, Santa-Cruz Biotechnology, sc-2001) were then added to each lysate and incubated 1h at 4°C (rotation). The beads were then washed 3 times with lysis buffer. Immunoprecipitated proteins were denatured in Laemmli buffer (20 µl of 3X; 150 mM Tris-HCl (pH 6.8), 300 mM DTT, 6% SDS, 0.3% bromophenol blue, and 30% glycerol) and boiled for 5 min. Proteins were resolved by 12% SDS–PAGE and analysed by immunoblotting.

ELISA

Retinal VEGFA concentration was measured by ELISA (MMV00, R&D Systems) according to the manufacturer's instructions, and normalized to the total cell protein content of each sample, as assessed by the Bradford assay. 661W cells were treated with GW9508 at 14μM for 6h before collection.

RT-PCR

mRNA from cells, retinas or laser-captured samples were extracted using the RNeasy kit (Qiagen) according to the manufacturer's protocol, and reverse transcribed to obtain cDNA libraries. PCR primers were designed using Primer Bank and NCBI Primer Blast software. Real time PCR were perfomed on a Lightcycler 480, Instrument II (Roche), using iTaq Universal SYBR Green Supermix kit (Bio-Rad). Gene expression was calculated relative to *cyclophilin A* using the ΔcT method. Primer sequences are reported in supplemental Table S3.

Immunoblotting

Retinal lysates (P16, 25-50 μg) were obtained from mice that had a confirmed neovascular phenotype in the contralateral eye; they were loaded on SDS-PAGE gels and electro-blotted onto a PVDF membranes. Membranes were then incubated with antibodies against total and ATG5 (Cell Signaling Technology), SIRT3 (Cell Signaling Technology), p62/SQSTM1 (Cell Signaling Technology), LC3B-II (Cell Signaling Technology), Lamin B1 (Cell Signaling Technology), Histone H3 (Cell Signaling Technology), ZKSCAN3 (Proteintech), phospho TFEB Ser142 and total TFEB (Cell Signaling Technology), total TFEB (Bethyl Laboratories), FFAR1 (Abcam), total and phospho p70S6K (Cell Signaling Technology), total and phospho MAPK 42/44 (ERK1/2) (Cell Signaling Technology), total and phospho GSK-3β and AKT (Cell Signaling Technology) and phospho Calcineurin Ser197 (Badrilla), Pan-Calcineurin A (Cell Signaling Technology). Western blot of β-actin or Tubulin (Cell Signaling) was used as a loading control. All antibodies were used according to the manufacturer's instructions. Revelation was done using ECL reagent from Thermofisher. Refer to Table S4.

Single-cell drop-seq

Single-cell suspensions were prepared from C57Bl/6 control retinas at P14, as reported (1), through successive steps of digestion (using papain solution; Worthington, LK003150), trituration and filtration to obtain a final concentration of 120 cells/µl. Droplet generation and cDNA libraries were performed as described in the Drop-seq procedure, and sequencing was done on Illumina NextSeq 500. We replicated the single cell results of Macosko et al., using the same biological samples (C57 retina), time points (P14) and methods (**Figure S1**) and confirmed identical clustering of the different retinal cell types with our reference sample. We therefore combined our retinal sample (r8) with WT replicates from Macosko et al (r3 and r5), for further comparison with *Vldlr*$^{-/-}$ retinas (3 biological replicates, P14, (~3000 cells/replicate).

Single cell RNA sequencing analysis

Unique molecular identifier (UMI) counts for WT and/or *Vldlr*$^{-/-}$ scRNAseq replicates were merged into one single Digital Gene Expression (DGE) matrix and processed using the "Seurat" package (2). Cells expressing less than 500 genes and more than 10% of mitochondrial genes were filtered out. Single cell transcriptomes were normalized by dividing by the total number of UMIs per cell, then multiplying by 10,000. All calculations and data were then performed in log space (i.e. log(transcripts-per-10,000 +1)). PCA analysis on the most variable genes in the DGE matrix identify 20 significant PC, which served as input for t-Distributed Stochastic Neighbor Embedding (tSNE) or Uniform Manifold Approximation and Projection (UMAP). To identify putative cell types on the embedded map, we used a density clustering approach and computed average gene expression for each of the identified cluster based on Euclidean distances. We then compared each of the different clusters to identify marker genes that were differentially expressed in the cluster. Transcriptomic differences between WT (n = 3801 cells) and *Vldlr*$^{-/-}$ (n = 5642 cells) cell types were statistically compared using a negative binomial model, visualization tools included Violin Plot, Split Dot Plot, Dot Plot and tSNE plot. Single-cell gene expression profiles from each separate cell type identified by scRNAseq were further analyzed using AUGUR (3), Gene Set Variation Analysis (4), pre-ranked Gene Set Enrichment Analysis (5, 6) and fast pre-ranked GSEA (7).

Oxygen consumption rates

Oxygen consumption rates (OCR) were measured using a Seahorse XF^e96 Flux Analyzer. Photoreceptor 661W cells were seeded in 96-well plates and cultured with BSA or BSA-Palmitate (0,2mM), in the presence of GW9508 (30μM, Cedarlane), HPβCD (1mM, Sigma-Aldrich), chloroquine (40μM, Sigma-Aldrich) or their respective vehicle for 8 hours. Cells were incubated in assay media (DMEM 5030 media, 12mM glucose, 1mM sodium pyruvate, 2mM glutamine, 10mM HEPES) with their respective drug treatments one hour prior to OCR measurements. Dependence on fatty acid β-oxidation was assayed by treating cells with Etomoxir (40μM; Sigma-Aldrich) 45 min prior to analysis. Oligomycin (1μM, Sigma-Aldrich), carbonyl cyanide-*p*-trifluoromethoxy-phenylhydrazone (FCCP; 2μM, Abcam) and rotenone-antimycin A (RAA; 0.5μM, Sigma-Aldrich) were added to cells to measure ATP production, maximal respiration, and non-mitochondrial respiration, respectively. Results were normalized using the number of cells in each well.

Laser micro dissection

Eyes from P16 mice were embedded in Frozen Section Media (Surgipath FSC22 Clear, Leica) and flash frozen immediately after enucleation. Cryosections (10µm) were collected on RNase-free polyethylene naphthalate glass slides (11505189, Leica). Sections were then stained for lectin (1:50 in 1mM $CaCl_2$) and dehydrated with 70%, 90% and 100% ethanol washes. Retinal vessels and layers were microdissected with a Leica LMD 6000 system (Leica Microsystems) and collected directly into RNA stabilizing buffer from the RNeasy Micro kit (Qiagen, Chatsworth, CA). RNA was extracted from microdissected tissues using the RNeasy Kit as described above (Qiagen), and real-time PCR was performed with the generated cDNA.

Super resolution confocal microscopy

Images of CAG-RFP-EGFP-LC3 retinas at P16 and photoreceptor 661W cells were acquired using the Lightning program of Leica TCS SP8 confocal microscope, using a 40 X (HP PL APO 40X/0.85 CORR CS, 0.11-) and 63X (HP PL APO 63X/1.40 OIL CS2) lenses. Image resolution of 1024x1204 and 3808x 3808 pixels were acquired and then deconvolved. Illumination was provided using a solid-state laser (20mW): 488 nm and 552 nm, as well as Diode (50mW): 405

nm laser. Slides were imaged at room temperature (~22˚C). Post-acquisition image analysis was performed with LAS-X and ImageJ software.

Autophagic flux quantification

Eyes from CAG-RFP-EGFP-LC3 and CAG-RFP-EGFP-LC3/*Vldlr*$^{-/-}$ mice P16 were fixed in 4% paraformaldehyde at room temperature 1h, cryoprotected with a sucrose gradient, and embedded using Surgipath FSC22 Clear (Leica). Cryosections (10μm) were prepared from a minimum of 6 mice per group and 1 section with corresponding volume of 0.226 μm^3 for each eye were analyzed. Autophagic flux was quantified using Imaris software and the 3D object colocalization tool.

Immunofluorescence

Cells were cultured to 80% confluence in 4-chamber slides (Nunc® Lab-Tek® II chambered coverglass, Sigma-Aldrich, cat Z734853) and pre-treated 17h with GW9508 (30[illegible]M, Cedarlane), MCT (0.4%) or vehicle. The cells were then washed with PBS-1X and starved 4 hours in serum-free DMEM, maintaining their respective drug treatments, before to be fixed 10 min in PFA4%. P16 eyes were collected and fixed 1h in PFA4%, at room temperature. Permeabilization was similarly performed for eyes and cells (20 min, 0.1% Triton X-100), and incubation was made in blocking buffer (20 min, PBS-BSA 5%) at room temperature. Immunostaining was done overnight with anti-Tfeb chip grade primary antibody (dilution 1/100 in PBS, Abcam #122910) or anti-p62/ SQSTM1 (Rodent specific, dilution 1/100, Cell Signalling # 23214S) and PNA-cy5 (dilution 1/100, Vector Lab # CL-1075-1), followed by secondary labeling using donkey anti-goat IgG Alexa fluor 488 (1 hour, 1/500 in PBS, ThermoFischer Scientific). Quantification of eGFP intensity or dots was measured using ImageJ software. Briefly, for TFEB nuclear localisation, confocal images were acquired using the SP8 confocal microscope (Leica) at 40X oil immersion under resonant scanner. Images were then loaded on ImageJ, color images were separated and analyzed using the region of interest (ROI) manager. ROI were selected based on Dapi signal (corresponding to nucleus) and were superposed onto eGFP signal image, defining nuclear eGFP intensity.

Vascular lesion quantification

Mice were euthanized at P16 using a saline-pentobarbital solution and eyes were enucleated and fixed for 1h in paraformaldehyde 4% at room temperature. Retinas were wash in PBS1X and dissected, then permeabilized in methanol (20 minutes at -20˚C) and stained overnight at room temperature with Isolectin B4 (1/100, Fluorescein labeled Griffonia (Bandeiraea) Simplicifolia Lectin I, Vector Lab # FL-1101-2) diluted in 1 mM CaCl2-PBS. Lectin-stained retinas were whole-mounted onto Superfrost/Plus microscope slides (Fisher Scientific) with the photoreceptor side up and embedded in Fluoro-gel (Fischer Scientific). For quantification of retina outer vascular lesions, 20 images of each whole-mounted retina were obtained at 10x magnification on a Zeiss AxioObserver.Z1 microscope and merged to form one image using AxioVision 4.6.3.0 software. Vascular lesion counts were analyzed by technicians blinded to the interventions using the SWIFT_MACTEL method, as described (8).

RNAscope *in situ* hybridization

Experiments were performed as per manufacturer instructions on fixed retinal cryosections (12μm) using the RNAscope 2.5 chromogenic assay (Duplex, ACD biotechne). WT and *Vldlr*$^{-/-}$ eyes at (P)16 were fixed in 4% PFA for 24 hours (4˚C). Eyes were then immersed in sucrose gradient (10 to 30%), frozen in optimal cutting temperature compound (Surgipath FSC22 Clear, Leica) and stored at -80 ˚C until use. *Ffar1* and *Tfeb* mRNA expression were targeted using probes designed by ACD-Biotechne. Retinas were then imaged with a Leica DME-6 microscope using a 20X dry objective (HC PL APO 20X/0.75 CS2) and analyzed with imageJ.

Triglyceride concentrations

Blood collected in EDTA tubes was centrifuged to obtain plasma from P16 WT and *Vldlr*$^{-/-}$ mice, fed MCT (40μl, once) or vehicle (collected 8 hours later). Triglyceride concentrations were measured using the Triglycerides kit and Analox GL5 analyser (Analox Instruments, UK) following manufacturer instructions.

Metabolite profiling

Retina and human vitreous metabolite extracts were analyzed using two liquid chromatography tandem mass spectrometry (LC-MS) methods to measure polar metabolites, as described previously (9, 10). Rapidly dissected WT and *Vldlr*$^{-/-}$ retinas (flash-frozen less than 1 min after euthanasia; 15 or 16 biological replicates) were homogenized in 80% methanol (8 µL/mg of tissue) containing the internal standards inosine-15N4, thymine-d4, and glycocholate-d4 (Cambridge Isotope Laboratories) using a TissueLyser II (Qiagen) bead mill for 4 minutes at 20 Hz. Samples were centrifuged (9,000 x g, 10 min, 4°C) and supernatants were collected for direct analysis using negative ion mode profiling and were 5-fold diluted using acetonitrile/methanol/formic acid (74.9:24.9:0.2 v:v:v) for positive ion mode profiling. For human vitreous samples, negative ion mode profiling samples were prepared from 30 µl extracted with 120 µl of 80% methanol containing inosine-15N4, thymine-d4 and glycocholate-d4 internal standards (Cambridge Isotope Laboratories; Andover, MA) while samples for positive ion mode profiling were prepared by extracting 10 µL with 90 µl of acetonitrile/methanol/formic acid (74.9:24.9:0.2 v:v:v). Negative ion mode data were acquired by injecting extracts (10 µL) onto a 150 x 2.0 mm Luna NH2 column (Phenomenex; Torrance, CA). The column was eluted at a flow rate of 400 µl/min with initial conditions of 10% mobile phase A (20 mM ammonium acetate and 20 mM ammonium hydroxide in water) and 90% mobile phase B (10 mM ammonium hydroxide in 75:25 v/v acetonitrile/methanol) followed by a 10 min linear gradient to 100% mobile phase A. MS analyses were carried out using electrospray ionization in the negative ion mode using full scan analysis over m/z 70-750 at 70,000 resolution and 3 Hz data acquisition rate. Additional MS settings were: ion spray voltage, -3.0 kV; capillary temperature, 350°C; probe heater temperature, 325 °C; sheath gas, 55; auxiliary gas, 10; and S-lens RF level 50. Postiive ion mode data were acquired by injecting extracts (10 µl) onto a 150 x 2 mm, 3 µm Atlantis HILIC column (Waters; Milford, MA). The column was eluted isocratically at a flow rate of 250 µl/min with 5% mobile phase A (10 mM ammonium formate and 0.1% formic acid in water) for 0.5 minute followed by a linear gradient to 40% mobile phase B (acetonitrile with 0.1% formic acid) over 10 minutes. MS analyses were carried out using electrospray ionization in the positive ion mode using full scan analysis over 70-800 m/z at 70,000 resolution and 3 Hz data acquisition rate.

Other MS settings were: sheath gas 40, sweep gas 2, spray voltage 3.5 kV, capillary temperature 350°C, S-lens RF 40, heater temperature 300°C, microscans 1, automatic gain control target 1e6, and maximum ion time 250 ms. Raw data were processed using TraceFinder 3.3 and 4.1 software (Thermo Scientific; Waltham, MA) and Progenesis QI (Nonlinear Dynamics; Newcastle upon Tyne, UK). For each method, metabolite identities were confirmed using authentic reference standards.

Human and mouse metabolomics profile were analyzed using MetaboAnalyst (11). Data was filtered based on interquartile range, then normalized using quantile normalization and auto-scaling. Outliers, detected by Random Forest algorithm, were removed and Principal component analysis (PLSDA) was performed on normalized data. Metabolite Set Enrichment Analysis was also performed using MetaboAnalyst. Correlated metabolites between mouse and human samples were identified and visualized using R/3.4.0 and the ShinyHeatmap package (12).

References

1. Macosko EZ, Basu A, Satija R, Nemesh J, Shekhar K, Goldman M, et al. Highly Parallel Genome-wide Expression Profiling of Individual Cells Using Nanoliter Droplets. *Cell.* 2015;161(5):1202-14.
2. Satija R, Farrell JA, Gennert D, Schier AF, and Regev A. Spatial reconstruction of single-cell gene expression data. *Nat Biotechnol.* 2015;33(5):495-502.
3. Skinnider MA, Squair JW, Kathe C, Anderson MA, Gautier M, Matson KJE, et al. Cell type prioritization in single-cell data. *Nature Biotechnology.* 2021;39(1):30-4.
4. Hänzelmann S, Castelo R, and Guinney J. GSVA: gene set variation analysis for microarray and RNA-Seq data. *BMC Bioinformatics.* 2013;14(7).
5. Subramanian A, Tamayo P, Mootha VK, Mukherjee S, Ebert BL, Gillette MA, et al. Gene set enrichment analysis: a knowledge-based approach for interpreting genome-wide expression profiles. *Proc Natl Acad Sci U S A.* 2005;102(43):15545-50.
6. Mootha VK, Lindgren CM, Eriksson K-F, Subramanian A, Sihag S, Lehar J, et al. PGC-1alpha-responsive genes involved in oxidative phosphorylation are coordinately downregulated in human diabetes. *Nat Genet.* 2003;34(3):267-73.
7. Korotkevich G, Sukhov V, and Sergushichev A. 2019.
8. Joyal J-S, Sun Y, Gantner ML, Shao Z, Evans LP, Saba N, et al. Retinal lipid and glucose metabolism dictates angiogenesis through the lipid sensor Ffar1. *Nature Medicine.* 2016;22(4):439-45.

9. Mills EL, Pierce KA, Jedrychowski MP, Garrity R, Winther S, Vidoni S, et al. Accumulation of succinate controls activation of adipose tissue thermogenesis. *Nature.* 2018;560(7716):102-6.
10. O'Sullivan JF, Morningstar JE, Yang Q, Zheng B, Gao Y, Jeanfavre S, et al. Dimethylguanidino valeric acid is a marker of liver fat and predicts diabetes. *J Clin Invest.* 2017;127(12):4394-402.
11. Xia J, and Wishart DS. Using MetaboAnalyst 3.0 for Comprehensive Metabolomics Data Analysis. *Curr Protoc Bioinformatics.* 2016;55:14.0.1-.0.91.
12. Khomtchouk BB, Hennessy JR, and Wahlestedt C. shinyheatmap: Ultra fast low memory heatmap web interface for big data genomics. *PLoS One.* 2017;12(5):e0176334.
